# Molecular insights into the Darwin paradox of coral reefs from the sea anemone Aiptasia

**DOI:** 10.1126/sciadv.adf7108

**Published:** 2023-03-15

**Authors:** Guoxin Cui, Migle K. Konciute, Lorraine Ling, Luke Esau, Jean-Baptiste Raina, Baoda Han, Octavio R. Salazar, Jason S. Presnell, Nils Rädecker, Huawen Zhong, Jessica Menzies, Phillip A. Cleves, Yi Jin Liew, Cory J. Krediet, Val Sawiccy, Maha J. Cziesielski, Paul Guagliardo, Jeremy Bougoure, Mathieu Pernice, Heribert Hirt, Christian R. Voolstra, Virginia M. Weis, John R. Pringle, Manuel Aranda

**Affiliations:** ^1^ Red Sea Research Center, Biological and Environmental Sciences and Engineering Division (BESE), King Abdullah University of Science and Technology (KAUST), Thuwal 23955-6900, Saudi Arabia.; ^2^ Department of Genetics, Stanford University School of Medicine, Stanford, CA 94305, USA.; ^3^ Core Labs, King Abdullah University of Science and Technology (KAUST), Thuwal 23955-6900, Saudi Arabia.; ^4^ Climate Change Cluster, University of Technology Sydney, Ultimo, NSW 2007, Australia.; ^5^ DARWIN21, Biological and Environmental Science and Engineering Division (BESE), King Abdullah University of Science and Technology (KAUST), Thuwal 23955-6900, Saudi Arabia.; ^6^ Department of Integrative Biology, Oregon State University, Corvallis, OR 97331, USA.; ^7^ Department of Biology, University of Konstanz, Konstanz 78457, Germany.; ^8^ Laboratory for Biological Geochemistry, School of Architecture, Civil and Environmental Engineering, École Polytechnique Fédérale de Lausanne, Lausanne, Switzerland.; ^9^ Department of Embryology, Carnegie Institution for Science, Baltimore, MD 21218, USA.; ^10^ Department of Marine Science, Eckerd College, St. Petersburg, FL 33711, USA.; ^11^ Centre for Microscopy Characterisation and Analysis, The University of Western Australia, Perth, WA, Australia.

## Abstract

Symbiotic cnidarians such as corals and anemones form highly productive and biodiverse coral reef ecosystems in nutrient-poor ocean environments, a phenomenon known as Darwin’s paradox. Resolving this paradox requires elucidating the molecular bases of efficient nutrient distribution and recycling in the cnidarian-dinoflagellate symbiosis. Using the sea anemone Aiptasia, we show that during symbiosis, the increased availability of glucose and the presence of the algae jointly induce the coordinated up-regulation and relocalization of glucose and ammonium transporters. These molecular responses are critical to support symbiont functioning and organism-wide nitrogen assimilation through glutamine synthetase/glutamate synthase–mediated amino acid biosynthesis. Our results reveal crucial aspects of the molecular mechanisms underlying nitrogen conservation and recycling in these organisms that allow them to thrive in the nitrogen-poor ocean environments.

## Teaser

Whole-organism nitrogen assimilation fueled by glucose from symbiotic algae enables corals to flourish in oligotrophic waters.

## INTRODUCTION

The ability of corals to build one of the planet’s most biodiverse and productive ecosystems in the nutrient-poor seawater of the subtropics and tropics, often referred to as “ocean deserts,” has both fascinated and puzzled scientists since it was first noted by Darwin (*1*). The foundation of these ecosystems is the symbiotic relationship between coral host animals and photosynthetic dinoflagellate algae (*2*, *3*) of the family Symbiodiniaceae (*4*), which live in specialized vacuoles (known as “symbiosomes”) inside the gastrodermal cells that line the gastric cavity of the host. The hosts and algae, together with a diverse assemblage of microorganisms, form metaorganisms known as holobionts (*5*). Algal photosynthesis provides fixed organic carbon for energy and biosynthesis and covers most of the hosts’ energy demands (*2*, *3*). However, the provision of organic carbon is not the only important function attributed to the algal endosymbionts. Nitrogen is one of the primary growth-limiting nutrients in coral reef ecosystems (*2*, *6*), and algae have been thought to be the main contributors to nitrogen acquisition and recycling (*7*–*11*) because of their high capacity for ammonium assimilation (*12*). However, some evidence has also suggested an active role for the host in nitrogen assimilation (*13*), a view supported by the recent realization that the host also has the enzymatic machinery to recycle ammonium via the glutamine synthetase/glutamate synthase (GS/GOGAT) system (*14*–*16*).

The importance of photosynthetically fixed carbon and of nitrogen assimilation and conservation for the ecological success and productivity of these metaorganisms is well established. However, we still do not know how fixed carbon is moved from algae to the various host cells as well as the respective contribution of the host and algae to nitrogen assimilation and conservation. Unraveling these mechanisms is critical for our understanding of holobiont functioning and ecological productivity. In this study, we used the sea anemone Aiptasia as a tractable system to experimentally investigate host-symbiont nutrient interactions in detail. Similar to corals, Aiptasia engages in an endosymbiotic relationship with dinoflagellates in the family Symbiodiniaceae (*17*–*19*), but it further offers the unique advantage of allowing comparisons between symbiotic and nonsymbiotic individuals (fig. S1).

## RESULTS

### Modulation of gene expression by tissue type and symbiotic state

**Fig. 1.**
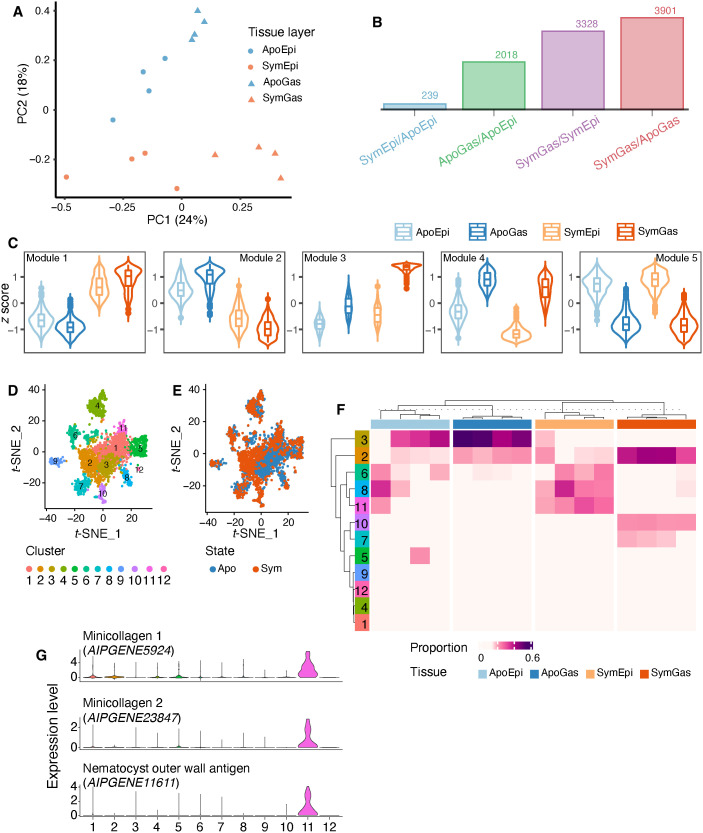
Transcriptomic profiles of tissues and host cells isolated from symbiotic and aposymbiotic Aiptasia. (**A**) PCA of LMD RNA-seq data generated from four biological replicates of each of four tissue types. Apo, aposymbiotic; Sym, symbiotic; Gas, gastrodermis; Epi, epidermis. (**B**) The numbers of differentially expressed genes (DEGs) identified in the pairwise comparisons (*q* < 0.05). (**C**) The five modules determined by hierarchical cluster analysis on the expression levels (scaled *z* scores) of the DEGs. (**D**) Clustering of 2698 Aiptasia cells into 12 clusters (each a different color) in *t*-distributed stochastic neighbor embedding (*t*-SNE) space based on their transcription profiles. Each dot represents one host cell. (**E**) The same 12 clusters with the symbiotic state of the source animal indicated for each cell. (**F**) The proportions of the 12 cell clusters in the bulk sequenced tissue-specific samples (four samples per tissue type). The cellular complexities of the tissue samples were characterized by MuSiC. (**G**) The expression patterns of three cluster 11 marker genes across all cells.

To investigate nutrient fluxes within the cnidarian-algal symbiosis in the context of the spatial organization of the holobiont, we first isolated gastrodermal and epidermal tissues from both symbiotic and aposymbiotic anemones (thus, four tissue types in total) using laser microdissection (LMD; fig. S2) and analyzed their transcriptomic profiles via RNA sequencing (RNA-seq) (data S1). A principal components analysis (PCA) showed samples clustered by both tissue layer (PC1: ~24% of the variance) and symbiotic state (PC2: ~18% of the variance) (Fig. 1A). Symbiosis induced extensive changes in gastrodermal gene expression and notable (although more limited) changes in epidermal gene expression (Fig. 1B). Using a multifactorial differential expression analysis including both tissue identity and symbiotic state, we identified 5414 gene expression signatures linked to one or both of these factors. Hierarchical clustering of these genes identified five modules showing distinct expression patterns (Fig. 1C and data S2): genes in modules 1 and 2 were symbiosis induced and repressed, respectively; genes in module 3 were symbiosis induced and gastrodermis specific; and genes in modules 4 and 5 were gastrodermis and epidermis specific, respectively.

We next performed functional enrichment analyses to identify the predominant functional categories of the genes within the modules (*P* < 0.05; fig. S3). Module 1 was enriched for genes associated with both carbon (table S1) and nitrogen (table S2) cycling (including at least one predicted glucose transporter and one predicted ammonium transporter) as well as protein posttranslational modifications (table S3), presumably reflecting the metabolic changes induced by symbiosis in both tissue layers. Module 2 was enriched for genes involved in food digestion (table S4), presumably reflecting the dependence of aposymbiotic animals on heterotrophic food sources. Expectedly, module 3 (symbiosis- and gastrodermis-specific) was enriched for functions related to the organization of the symbiosome (table S5) and the transport of various key metabolites, such as glucose (table S6) and cholesterol (table S7), whereas the gastrodermis-specific module 4 included many digestion-related genes (table S8), and the epidermis-specific module 5 was enriched for genes involved in cnidocyte function (table S9) and responses to mechanical stimuli (table S10).

Given the central importance of the GS/GOGAT cycle in ammonium assimilation and the strong symbiosis-induced up-regulation of the Aiptasia genes for both enzymes at the whole-organism level (fig. S4A) (*8*, *14*–*16*), we were surprised that these genes did not appear in either module 1 or module 3. However, as we also did not find any appreciable difference between gastrodermis and epidermis in the expression levels of either GS or GOGAT (fig. S4B), the most parsimonious interpretation is that the tissue-specific expression data are somehow misleading in this case (see Discussion) and that GS and GOGAT participate in enhanced ammonium assimilation by both major tissues of symbiotic anemones, consistent with the transporter localization and nanoscale secondary ion mass spectrometry (NanoSIMS) data presented below.

### Cell type–specific responses to symbiosis

Although many of the differentially expressed genes (DEGs) identified in the analysis of tissue-specific expression had been reported previously to be symbiosis regulated (*14*, *15*, *20*), our analysis began to provide the spatial resolution needed to investigate their functions further. To gain higher-resolution spatial information, we next performed single-cell RNA-seq on isolated cells using the 10x Genomics platform. We retrieved gene expression information from 2698 cells, of which 1453 originated from aposymbiotic and 1245 from symbiotic anemones. Following *t*-distributed stochastic neighbor embedding analysis, we grouped the cells into 12 clusters with distinct gene expression profiles (Fig. 1D) and identified potential cell type marker genes (adjusted *P* < 0.01 and average fold change > 2; data S3). Most of these clusters were shared between symbiotic and aposymbiotic animals, but some clusters were largely specific to one symbiotic state (Fig. 1E and fig. S5).

To investigate the tissue origins of the cells in the 12 clusters, we integrated our single-cell and tissue-specific data by performing a deconvolution analysis using MuSiC (*21*), which indicated that five of the cell clusters (1, 4, 5, 9, and 12) were present at low abundance (<10% of the total cells) in all of the tissue samples (Fig. 1F; see Discussion). The other seven clusters exhibited tissue- and/or symbiotic state–specific associations (Fig. 1F). Cluster 11 was present exclusively in the epidermis, and its highly expressed marker genes are cnidocyte specific, as identified in other cnidarian species (Fig. 1G and table S11) (*22*–*24*), so that this cluster appears to represent cnidocytes. In contrast, clusters 2, 7, and 10 were highly represented in the gastrodermal samples, with the latter two groups being specific to symbiotic animals (Fig. 1F). Many of the highly expressed marker genes for these clusters (table S12) had been identified previously as displaying gastrodermis-specific expression in the sea anemone *Nematostella* (*25*), the stony coral *Stylophora* (*26*), and/or the soft coral *Xenia* (*23*), consistent with a gastrodermal origin for these cells.

We then further examined the functions of the putative symbiotic gastrodermal cells through a gene set enrichment analysis of their marker genes (data S4 and S5). Cluster 10 marker genes were enriched for ones associated with lysosomal organization and function (table S13), glucose transport and its positive regulation (table S14), and cholesterol homeostasis (table S15), whereas cluster 7 marker genes were enriched for functions associated with the extracellular matrix, cell adhesion, and cell-cell signaling (table S16). Thus, cluster 10 appears to be symbiotic cells, whereas cluster 7 may contain gastrodermal cells that are free of algal cells.

### Symbiosis induces elevated expression and relocalization of glucose and ammonium transporters

**Fig. 2.**
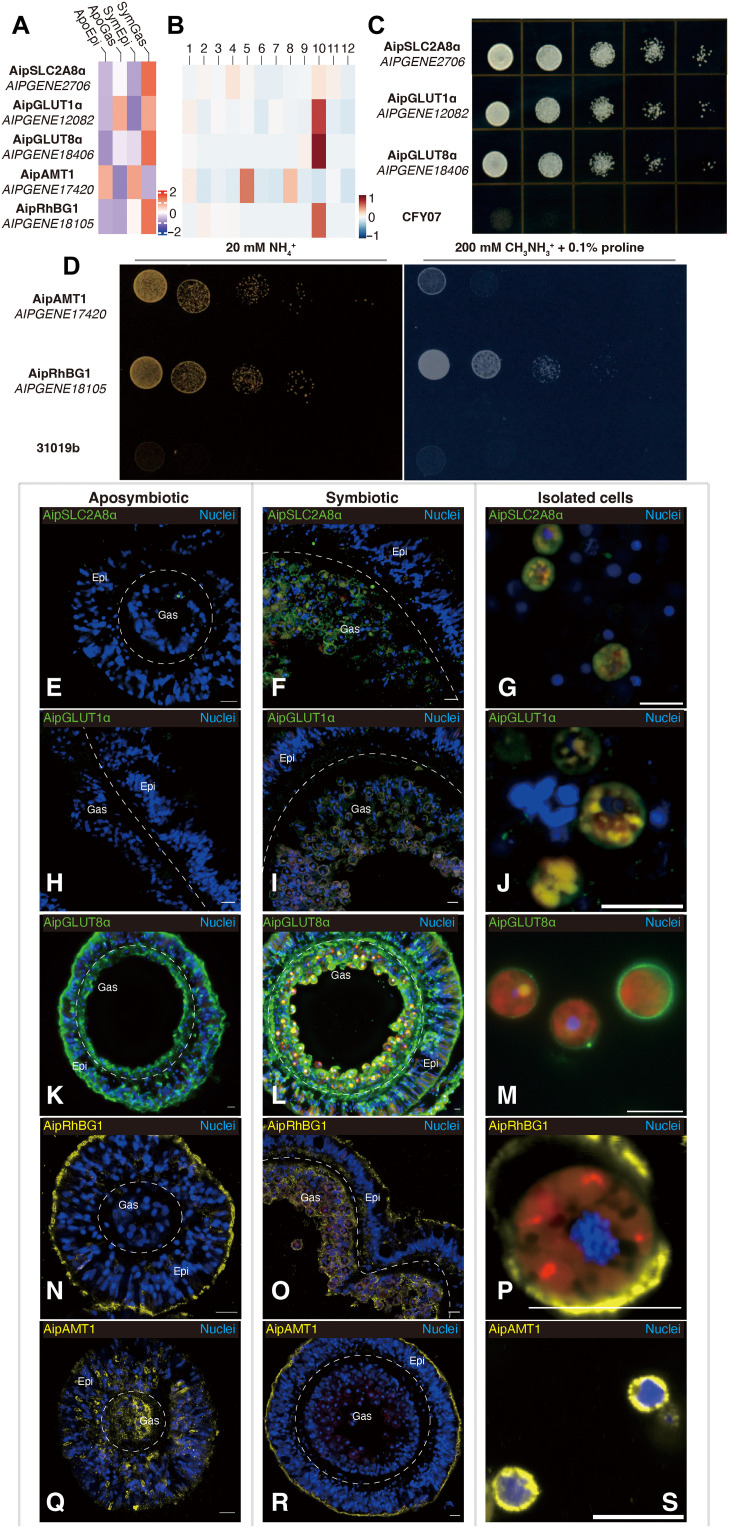
Altered expression and relocalization of glucose and ammonium transporters during symbiosis. (**A** and **B**) Expression patterns of mRNAs for glucose and ammonium transporters at the tissue (A) and cell (B) levels. (**C**) Rescue experiments on yeast mutant CFY07 (lacking all sugar transporters; see Methods) expressing one of the putative Aiptasia glucose transporters and spotted in a dilution series on medium containing glucose as the sole carbon source. (**D**) Rescue experiments on yeast mutant strain 31019b (lacking all ammonium transporters; see Methods) expressing Aiptasia AipAMT1 or AipRhGB1. The plate on the left contained 20 mM ammonium as the sole nitrogen source. The plate on the right contained 0.1% proline as a nitrogen source plus 200 mM of the toxic ammonium analog methylammonium. (**E** to **S**) Immunofluorescence staining of glucose (E to M) and ammonium (N to S) transporters in tissue sections of aposymbiotic (E, H, K, N, and Q) or symbiotic (F, I, L, O, and R) anemones, as well as in cells isolated from symbiotic animals (G, J, M, P, and S). Scale bars, 10 μm.

To explore further the tissue- and cell-specific incorporation of carbon and nitrogen, we focused on the major glucose and ammonium transporters. At least six putative glucose transporters and two putative ammonium transporters showed significant changes in tissue- and/or cell-specific expression in response to symbiosis (Fig. 2, A and B, and tables S1, S2, S6, S14, and S17), so we examined the functions and localizations of five of these transporters in more detail.

First, we conducted rescue experiments in yeast mutants to test the putative transporter activities of the gene products. Each one rescued the appropriate yeast mutant (*27*, *28*), allowing its growth on the relevant selective medium (Fig. 2, C and D, left), verifying that each had the predicted glucose or ammonium transporter activity.

We next generated antibodies specific for each of these five transporters (see Methods; fig. S6 and table S18) and used these antibodies to examine their localizations by immunofluorescence staining. AipSLC2A8α (Fig. 2, E to G, and figs. S7 and S8, A to C) and AipGLUT1α (Fig. 2, H to J, and figs. S7 and S8, D to F) were detected primarily or exclusively in symbiotic gastrodermis and in isolated symbiotic cells. For AipGLUT1α (*AIPGENE12082*), the apparent discrepancy between these results and those on tissue-specific transcript levels (Fig. 2A and table S17) may reflect a misleading feature of the latter, given that *AIPGENE12082* transcript levels were substantially up-regulated in the putatively gastrodermal cluster 10 cells in the single-cell analysis (Fig. 2B and table S14; see Discussion). Their localization patterns suggest that these transporters might serve mainly to move photosynthetically produced glucose (*29*) across the symbiosome membrane into the host-cell cytoplasm and/or from the symbiotic cells to the rest of the organism. In contrast, AipGLUT8α, despite its high apparent tissue (Fig. 2A and tables S6 and S17) and cell (Fig. 2B) specificity as seen by RNA-seq, was detected both in the outer (seawater-facing) surface of the epidermis and the inner (body cavity–facing) surface of the gastrodermis in aposymbiotic anemones (Fig. 2K and fig. S8G), suggesting that, in such animals, it might have a role in the scavenging of environmental glucose. In symbiotic anemones, however, this localization was largely replaced by one in which the peripheries of the gastrodermal cells, or perhaps their symbiosomes (the images do not have sufficient resolution to tell), were heavily stained, along with substantial staining of the gastrodermis-epidermis boundary (Fig. 2, L and M, and fig. S8, H and I), suggesting a role for AipGLUT8α also in the dissemination of photosynthetically produced glucose.

Localization of the two ammonium transporters also changed in response to symbiosis. AipRhBG1 was observed primarily at the outer surface of the epidermal cells in aposymbiotic animals (Fig. 2N and fig. S8J), suggesting a role in the excretion of excess ammonium in the heterotrophic animals. In contrast, in symbiotic animals, although the protein was still observed in the outer layer of the epidermis, it was now most prominent around the gastrodermal cells and along the gastrodermis-epidermis boundary (Fig. 2, O and P, and fig. S8, K and L), suggesting that, under these conditions, it functions in the uptake of ammonium for both animal and algal use. Consistent with the hypothesis that AipRhBG1 can transport ammonium both out of and into Aiptasia cells, yeast cells expressing AipRhBG1 as their sole ammonium transporter were relatively resistant to the toxic ammonium analog methylammonium (Fig. 2D, right), suggesting that this compound did not accumulate to high levels in the cells (*30*). In contrast, yeast cells expressing only AipAMT1 were much more sensitive to the drug (Fig. 2D, right), suggesting that this transporter functions only in ammonium uptake. Consistent with this hypothesis, immunofluorescence staining found AipAMT1 diffusely localized in aposymbiotic animals (Fig. 2Q and fig. S8M) but concentrated in the outer layer of the epidermis in symbiotic animals (Fig. 2, R and S, and fig. S8, N and O).

### Effects of metabolism and symbiosis on gene expression and protein localization

**Fig. 3.**
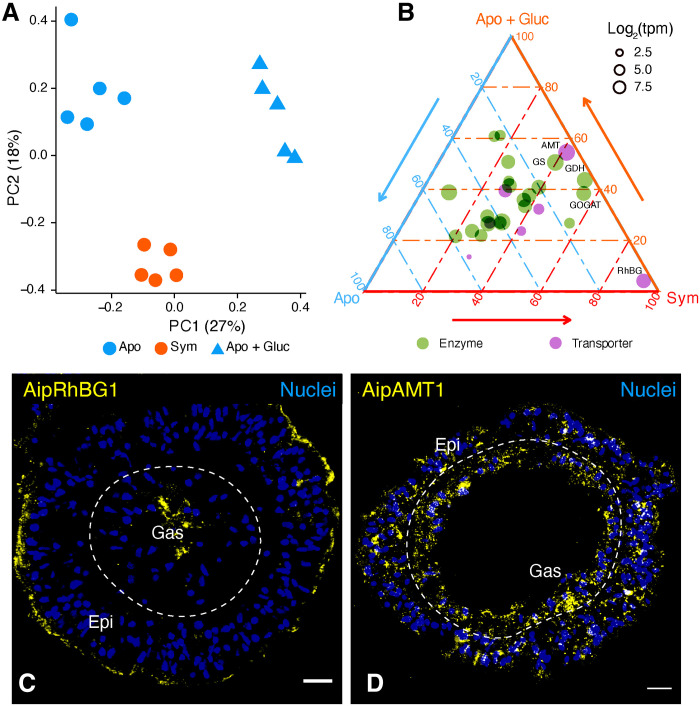
Expression changes of nitrogen metabolism genes in response to glucose supplementation. (**A**) PCA of whole-animal Aiptasia RNA-seq data from an experiment comparing expression in aposymbiotic anemones with added glucose (Apo + Gluc) to that in aposymbiotic and symbiotic anemones without added glucose. (**B**) Ternary plot showing relative expression levels of genes associated with “nitrogen-metabolism pathway” (Kyoto Encyclopedia of Genes and Genomes, ko00910) in Aiptasia. Each dot represents a gene with coordinates representing the relative proportion of its expression level in each condition relative to its overall expression across the three conditions. tpm, transcripts per million. (**C** and **D**) Immunofluorescence staining of AipRhGB1 (C) and AipAMT1 (D) in aposymbiotic Aiptasia supplied with glucose. Scale bars, 10 μm.

We next asked whether the symbiosis-specific changes in gene expression and transporter localization were triggered simply by the increased availability of glucose when algae are present or by some other aspect of algal presence. The data presented above suggested that both photosynthetically derived glucose and ammonium are distributed throughout the symbiotic animals, which might, in turn, induce a high expression of the central GS/GOGAT ammonium assimilation machinery in both epidermal and gastrodermal cells. To test this hypothesis, we provided aposymbiotic anemones with supplemental glucose and analyzed gene expression changes in comparison to aposymbiotic and symbiotic anemones without added glucose. We found that glucose treatment significantly altered the whole-organism transcriptomic profiles (Fig. 3A). In particular, the expression levels of the AipAMT1 ammonium transporter and of several key nitrogen metabolism enzymes, including GS and GOGAT, were essentially the same in symbiotic and glucose-treated aposymbiotic animals (Fig. 3B and table S19). Thus, some aspects of a shift toward organism-wide assimilation of ammonium in symbiotic anemones appear to be a direct response to the increased availability of glucose.

However, other observations suggest a more complicated picture. First, AipRhGB1 mRNA levels were not increased in glucose-treated aposymbiotic anemones (Fig. 3B). Moreover, immunofluorescence staining of AipRhGB1 and AipAMT1 in the glucose-treated aposymbiotic anemones showed no obvious change in the localization of either protein (Fig. 3, C and D, and fig. S9; cf. Fig. 2, N and Q). Thus, both the symbiosis-induced increase in AipRhBG1 expression and the changes in the localization of both ammonium transporters (see above) appear to depend on the actual presence of the algae. Thus, although the metabolic response to assimilate ammonium via the GS/GOGAT pathway appears to depend only on the increased availability of glucose, a separate algae-dependent mechanism appears to exist for the regulation of nitrogen provision to the algae.

### Coordinated incorporation of carbon and nitrogen in both gastrodermis and epidermis of symbiotic animals

**Fig. 4.**
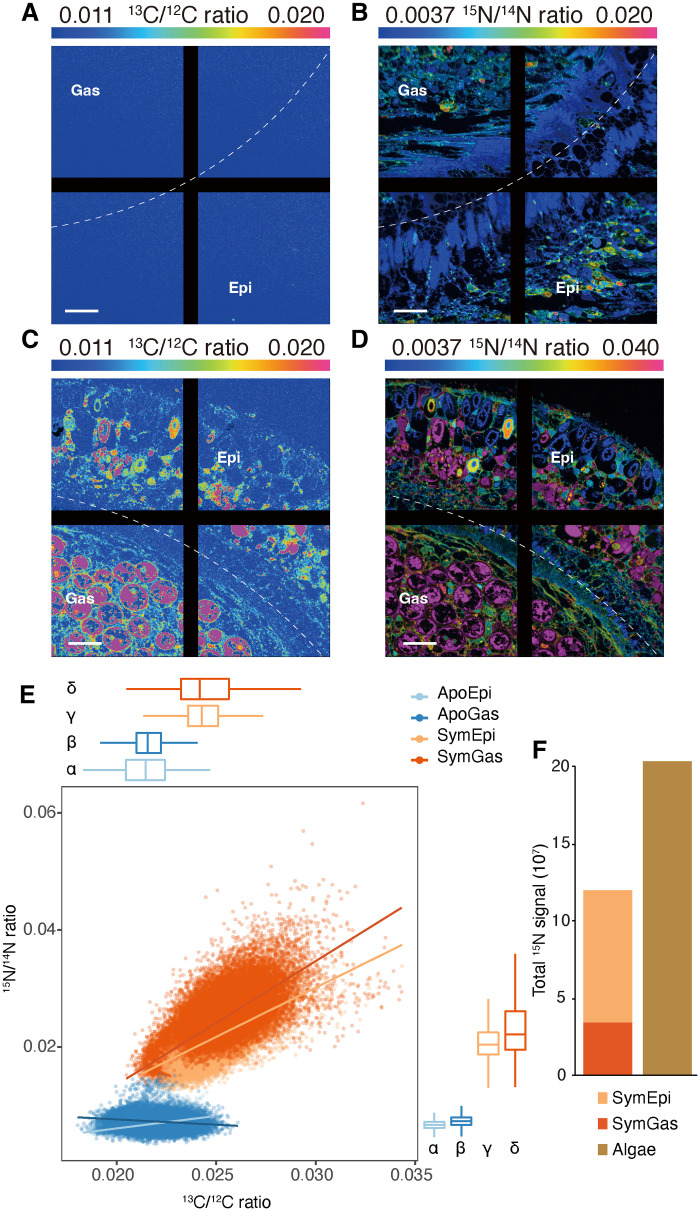
Carbon and nitrogen incorporation into gastrodermis and epidermis in aposymbiotic and symbiotic Aiptasia. (**A** to **D**) Representative images showing the distributions of ^13^C/^12^C (A and C) and ^15^N/^14^N (B and D) ratios in aposymbiotic (A and B) and symbiotic (C and D) Aiptasia. The ratios are displayed as hue-saturation-intensity images in which blue indicates the natural abundance isotope ratio, with a shift toward magenta indicating an increasing ^13^C or ^15^N incorporation level. Scale bars, 10 μm. The black lines are a feature of the NanoSIMS display without biological significance. (**E**) The correlations between ^13^C/^12^C and ^15^N/^14^N ratios in epidermis and gastrodermis from aposymbiotic and symbiotic anemones. ^13^C/^12^C and ^15^N/^14^N ratios were quantified at the pixel level (spatial resolution, 88 nm) for regions of interest (see fig. S10) across whole tentacle sections. Dots in the scatterplot represent the individual bins calculated, and the trend lines were estimated based on a generalized linear model. Box-and-whisker plots show the distributions of ^13^C/^12^C and ^15^N/^14^N ratios for each tissue layer; Greek letters indicate statistically significant (*P* < 0.001) differences between tissue layers as calculated using one-way analysis of variance (ANOVA) with Games-Howell post hoc tests. (**F**) Total absolute ^15^N signal in animal tissue layers and algal cells of symbiotic anemones. The marking method shown in fig. S10 was used to separate animal tissue and algal cells for each section. The total ^15^N signal was then calculated by summing raw pixel values collected from the ^15^N channel as shown in fig. S10B.

The data presented above suggest that symbiosis induces the organism-wide distribution of photosynthetically produced glucose and the assimilation of ammonium. To test this idea further, we incubated animals with ^13^C bicarbonate and ^15^N ammonium and quantified the assimilation of carbon and nitrogen in the gastrodermis and epidermis using NanoSIMS. The ^13^C-labeled glucose should provide both gastrodermal and epidermal cells with both adenosine triphosphate and carbon backbones required for the assimilation of ammonium into amino acids, nucleic acid bases, and other compounds. Thus, we expected a strong spatial correlation of the ^13^C and ^15^N signals in both major host tissues in symbiotic animals. As expected, the assimilation of both ^13^C and ^15^N was low in both tissues of aposymbiotic animals (Fig. 4, A and B), and ^13^C and ^15^N assimilations were not well correlated (Fig. 4E). In contrast, symbiotic anemones incorporated significantly more of both isotopes in both major tissue layers (Fig. 4, C and D). We also found a strong and statistically significant spatial correlation of the ^13^C and ^15^N signals in symbiotic anemones (at a spatial resolution of 88 nm; Fig. 4E and fig. S10), suggesting that these elements might be co-incorporated into similar biosynthetic products in both major tissue layers.

To further assess the contribution of ammonium assimilation by the host tissue layers to the overall nitrogen assimilation by the holobiont, we extracted absolute ^15^N signals from animal tissues and algal cells (marking method; fig. S10B). The host tissues (excluding algae) contained 35 to 41% of the total ^15^N signal in symbiotic anemones (Fig. 4F), and no statistically significant difference for the absolute ^15^N signals was found between host and algae (paired *t* test, *P* = 0.41). The epidermis actually incorporated significantly more ^15^N than the corresponding gastrodermis (paired *t* test, *P* = 0.02). Thus, both gastrodermis and epidermis of the host contribute substantially, on a par with the algae, to the overall holobiont nitrogen incorporation.

## DISCUSSION

For a century and a half after it was first noted by Darwin (*1*), the apparent paradox of highly productive and species-rich coral reefs thriving in nutrient-poor ocean waters remained a mystery. The first major step in resolving this paradox was the discovery that most of the organic carbon required by the coral animals for energy and biosynthesis is provided through photosynthesis by their endosymbiotic dinoflagellate algae (*2*, *3*). A second major step was the recognition that the problem of limiting nitrogen in marine environments (*2*, *6*) was solved, in part, by nitrogen conservation and/or recycling within the coral holobiont (*8*, *11*). However, these pioneering studies left many critical questions unanswered. In what form, and how, is the photosynthetically fixed carbon passed from the algae to the host gastrodermal cells and then distributed to the other host cells and tissues? How is excess nitrogen (when present) disposed of, and how is available nitrogen acquired from the environment when needed? What are the respective roles of the animal and its algal partner in the conservation and/or recycling of nitrogen within the holobiont? In addition, what aspect(s) of algal presence trigger the adjustments in gene expression and metabolism that the host must make for an effective symbiosis? In this study, we addressed these questions through a combination of whole-animal, tissue-specific, and single-cell analyses of gene expression levels; functional characterization and immunolocalization of glucose and ammonium transporters; NanoSIMS analysis to localize the sites at which new carbon and new nitrogen are incorporated into the holobiont; and experiments in which exogenous glucose was provided to aposymbiotic animals to mimic the supply of glucose from the algae in symbiotic animals. Note that beyond their use here in helping to answer the questions noted above, the extensive datasets on tissue-specific and cell type–specific gene expression should also be a valuable resource for a variety of future studies. In this regard, however, we do also note the caveat that the (mostly) tentacle-derived data on tissue-specific expression may not in all cases reflect the situation in the rest of the animal.

Previous experiments using rapid tracking of the fate of ^13^C bicarbonate supplied to symbiotic Aiptasia indicated that newly fixed carbon is transferred from the algae to the host gastrodermal cells primarily as glucose (*29*). This hypothesis has been further supported by the increased concentration of free glucose in symbiotic anemones (*31*). We have now provided further support for this hypothesis by showing that the expression of at least six glucose transporters is up-regulated in symbiotic relative to aposymbiotic gastrodermal cells. Moreover, the localizations of three such transporters examined are consistent with their putative roles in the transport of glucose across the symbiosome membrane into the cytoplasm of the gastrodermal cells, across the plasma membranes of the gastrodermal cells into the adjacent tissue, or both. Although the resolution of the immunofluorescence images is not sufficient to discriminate among these possibilities, the hypothesis that glucose is trafficked from the gastrodermal cells to the epidermal (and other) cells is also supported by the observations (i) that at least two of the glucose transporters are also significantly up-regulated in symbiotic relative to aposymbiotic epidermal cells and (ii) that at least one of the glucose transporters (AipGLUT8α) appears to be localized, in part, to the gastrodermis-epidermis boundary.

Similarly, our analysis of the expression and localization of ammonium transporters suggests that, in animals with few or no algal symbionts, the excess ammonium generated by heterotrophic metabolism (*8*, *13*, *16*, *32*) is excreted to the environment at least in part through the bidirectional transporter AipRhBG1 in the epidermal cell outer membranes. In contrast, when the supply of algal-derived glucose allows abundant incorporation of ammonium into organic compounds, thus reducing intracellular ammonium concentrations (*33*), both the unidirectional transporter AipAMT1 and a substantial fraction of the AipRhBG1 are found at the outer surface of the epidermis, and thus in a position to take up environmental ammonium. In addition, another substantial fraction of the highly up-regulated AipRhBG1 is found around the gastrodermal cells and at the epidermis-gastrodermis boundary, as also observed in the coral *Acropora yongei* (*34*), and thus in a position to distribute both retained and acquired ammonium from the epidermis to the gastrodermal cells and their resident algae.

Despite longstanding appreciation of the importance of conservation and/or recycling of nitrogen for symbiotic cnidarians (*8*, *11*, *13*, *15*, *16*, *32*, *35*), it has remained unclear which elements of the holobiont are responsible for these activities. Our results suggest that not only the algae but also both major host tissues are involved. First, our transporter studies suggest that algal-derived glucose and environmental ammonium are both available to both the gastrodermal and epidermal cells of the host and to the algae. Second, in agreement with earlier studies of GS enzymatic activities (*8*) and of GS and GOGAT mRNA levels (*14*–*16*), we found both mRNAs to be highly up-regulated at the whole-organism level in symbiotic animals, while no significant difference was found between gastrodermis and epidermis in this regard, suggesting that both major host tissues participate in ammonium incorporation by the GS/GOGAT system during symbiosis. Last, the NanoSIMS data show a robust and highly coordinated incorporation of both ^13^C and ^15^N in both major tissues of the host, with a total incorporation on a par with that in the algae. These findings are consistent with a recent metabolomic study showing that carbon and nitrogen are significantly co-integrated into amino acids by different symbiotic cnidarians, including Aiptasia and the coral *Stylophora pistillata* (*33*). Moreover, the observation that the total ^15^N incorporation was actually greater in the epidermis than in the gastrodermis argues against the possibility that the organic nitrogen compounds are all synthesized in the algae and then passed to the host.

It had seemed possible that the provision of glucose by the algae to the host was all that was needed to trigger the entire suite of changes in gene expression, metabolic function, and cellular organization that distinguish a symbiotic anemone from an aposymbiotic one. When we provided exogenous glucose to aposymbiotic animals, some changes in gene expression (and presumably in the metabolic pathways governed by those gene products) mimicked those in symbiotic animals. Most notably, GS and GOGAT were up-regulated similarly in the two cases, indicating that the metabolic response to incorporate more ammonium only requires an abundant supply of glucose to be triggered. However, many other changes in gene expression that are seen in symbiotic animals (notably the up-regulation of the AipRhBG1 ammonium transporter), as well as the relocalization of ammonium transporters, were not reproduced in the aposymbiotic animals provided with exogenous glucose. Thus, the algae must provide at least one other signal to the host that promotes the additional responses needed for an effective symbiosis. Disruption of such a signal(s) under stress conditions might affect the expression (*36*) and/or localization of symbiosis-associated nutrient transporters and thus disrupt the coordinated incorporation of carbon and nitrogen (*37*), leading to the breakdown of nutrient cycling and symbiosis. Hence, it will be of great interest to determine the nature of this signal(s).

In summary, we have used a combination of genomic, genetic, cell biological, physiological, and biophysical methods to clarify several previously obscure but very important aspects of the interaction between the host and the algae in the cnidarian-dinoflagellate symbiosis. Most notably, our data indicate that both major host tissues and the symbiotic algae all participate in the critical conservation and recycling of nitrogen, a resource that is typically limiting for growth in the coral reef environment.

## METHODS

### Animals and maintenance

Throughout this study, “seawater” refers to autoclaved, filtered seawater from the Red Sea. All experiments in this study used individuals of the sea anemone Aiptasia (sensu *Aiptasia pallida*, *Exaiptasia pallida*, or *Exaiptasia diaphana*) clonal strain CC7 (*38*) maintained in seawater. To generate anemones free of algal endosymbionts, animals were treated by cold shock at 4°C for 4 hours, followed by ~30 days of treatment in 50 μM diuron (Sigma-Aldrich) with daily water changes (*15*). At the end of the treatment, all anemones were individually inspected by fluorescence microscopy to confirm the absence of algal chlorophyll. A subset of these aposymbiotic anemones was then inoculated with the symbiotic *Breviolum minutum* algal strain SSB01 (*39*) and cultured until algal numbers were stable. Both symbiotic and aposymbiotic anemones were kept on a 12-hour light:12-hour dark cycle with ~40 μmol photons m^−2^ s^−1^ of photosynthetically active radiation and fed with freshly hatched brine shrimp (*Artemia salina*) approximately three times per week with seawater changes the day after feeding.

Eight small tanks (~500 ml) were set up for this study, with four tanks for symbiotic and four for aposymbiotic anemones. All tanks contained anemones of similar size (pedal disc of ~0.5 cm in diameter) and were maintained in the same incubator for the entire duration of the study. Before any sampling, the animals were not fed for 3 days to prevent contamination with genetic material from the brine shrimp. Aposymbiotic anemones were confirmed to be free of algae by fluorescence microscopy immediately before sampling for experiments.

### Laser microdissection

One anemone from each of the eight 500-ml tanks was collected at 11:00 a.m. (~6 hours into the light period), immediately snap-frozen in liquid nitrogen, embedded in the Tissue Freezing Medium (Electron Microscopy Sciences), and stored at −80°C until cryosectioning. The cryostat (CM3050 S, Leica) was prechilled to a chamber temperature of −23°C, and samples were equilibrated to the chamber temperature for 20 min and then sectioned at a thickness of 8 μm. Sections were then transferred onto precooled ribonuclease (RNase)–free polyester membrane metal frame slides (Leica) and air-dried for 5 min in a RNase-free fume hood.

Gastrodermal and epidermal cell layers were identified in sections (mostly from tentacles) at both ×10 and ×20 magnifications using a Leica LMD 6000 microscope with Leica filter cubes B/G/R and A. Each region of interest was traced individually using the LMD software and dissected using an ultraviolet laser beam. The dissected pieces were collected in CapSure Macro LCM Caps (Thermo Fisher Scientific) containing 40 μl of RNA extraction buffer from the Arcturus PicoPure RNA Isolation Kit (Thermo Fisher Scientific). The harvested cells were lysed by incubation at 42°C for 30 min, vortexed briefly, and then kept at −80°C until further processing.

### Glucose supplementation

To test the effect of exogenous glucose on gene expression, we compared the transcriptomic profiles of glucose-supplemented aposymbiotic anemones with those of symbiotic and aposymbiotic animals without glucose supplementation. Experiments were performed using three wells in each of five six-well plates; each well contained 8 ml of seawater. Each plate contained one symbiotic and two aposymbiotic anemones in separate wells, and glucose was added to one of the wells containing an aposymbiotic anemone at a final concentration of 10 mM. Seawater was changed every 2 days, and a new glucose dose was added after each water change. To avoid batch effects, the five plates, representing five biological replicates, were processed simultaneously. The whole experiment lasted for 11 days, with anemones being collected on the last day at 11:00 a.m., ~6 hours into the light period. The collected animals were snap-frozen in liquid nitrogen and kept at −80°C until further processing for whole-animal RNA-seq analysis.

### Isolated tissue and whole-animal RNA-seq

For the LMD-isolated tissue samples, total RNA from each cell lysate was extracted using the Arcturus PicoPure RNA Isolation Kit following the protocol for use with CapSure Macro LCM Caps. The quality of each RNA sample was assessed on an Agilent 2100 Bioanalyzer using the Agilent RNA 6000 Pico Kit. cDNA was synthesized using the Ovation RNA-seq system V2 kit (NuGen) following the manufacturer’s instructions. The amplified cDNA was fragmented to ~200 base pairs (bp) by shearing using a Covaris ultrasonicator according to the manufacturer’s suggested protocol. The fragmented cDNA was used to generate multiplexed sequencing libraries (mean insert size of 200 to 250 bp) using the NEBNext Ultra II DNA Library Prep Kit for Illumina sequencing. The samples were pooled and sequenced on four lanes of an Illumina HiSeq 2000 to generate paired-end reads.

For samples collected from the glucose supplementation experiment, the total RNA was extracted using the RNeasy Mini Kit (QIAGEN) following the manufacturer’s protocol for extraction from animal tissues. RNA quality was then assessed as described above. RNA-seq libraries were prepared using the TruSeq RNA Library Preparation Kit v2 (Illumina) according to the manufacturer’s instructions. All 15 libraries were then pooled and sequenced on an S1 flow cell with an Illumina NovaSeq 6000.

RNA-seq reads were mapped against the revised Aiptasia gene models (*40*) to quantify their expression levels using kallisto v0.44.0 (*41*). For the LMD-isolated tissue samples, analysis of differential gene expression was performed using sleuth v0.29.0 (*42*) with two schemes. First, four pairwise comparisons (SymGas versus ApoGas, SymEpi versus ApoEpi, SymGas versus SymEpi, and ApoGas versus ApoEpi) were performed to identify the main drivers of gene expression changes. Second, multiple-factor analysis including both tissue source and symbiotic state was applied to determine their effects on the expression profiles. The DEGs identified in the latter scheme were further clustered into five modules based on hierarchical clustering. Gene Ontology term enrichment analysis was conducted on the genes in these modules using topGO (*43*) as described previously (*15*).

In the glucose supplementation experiments, differential expression analysis was done only with the pairwise scheme. Expression levels of DEGs identified from each pairwise comparison were expressed as transcripts per million. The gene expression levels in each condition were compared and visualized using a ternary plot (*44*).

### Single-cell RNA-seq, data clustering, and marker identification

Single-cell RNA-seq was performed to determine the cell type–specific gene expression profiles for all Aiptasia cell types. One symbiotic and one aposymbiotic anemone were rinsed with 10 ml of phosphate-buffered saline (PBS; Merck) and dissected in 5 ml of the same buffer containing Liberase (100 μg/ml; Roche, SKU 5401119001) at 23°C for 1 hour. Each cell suspension was filtered through a 40-μm Falcon cell strainer (Thermo Fisher Scientific), adjusted to a density of ~2000 cells/μl, and run directly on the 10× Chromium system (10× Genomics). Single-cell libraries were generated using the 10× Chromium Single-Cell 3′ Reagent Kit V3 according to the manufacturer’s instructions. Library size distribution and concentration were determined using the Agilent Bioanalyzer with the High Sensitivity DNA kit and the QuantStudio 3 real-time polymerase chain reaction system (Thermo Fisher Scientific) with the KAPA DNA quantification kit (Roche), respectively. The libraries were sequenced using an Illumina HiSeq 4000 to generate paired-end reads.

Reads from single-cell RNA-seq were processed using kallisto v0.46.2 (*41*) and BUStools v0.40.0 (*45*) with a wrapper for single-cell data (*46*). The resulting expression matrices were then read into R and analyzed subsequently using Seurat v3.2.0 (*47*). Cells with <200 unique molecular identifiers were removed from further analyses.

The data derived from different samples were integrated using Seurat with the batch effect removal workflow. Briefly, the 2000 genes with the highest dispersion in each sample were identified and used as anchor features for data integration. The first 30 dimensions were included as a neighbor search space to find connections between samples. The cell clusters were classified at a resolution of 0.9 following the Leiden algorithm (*48*). The marker genes for each cell cluster were identified using a Wilcoxon rank sum test (adjusted *P* < 0.05), the default test specified in the FindAllMarks function implemented in Seurat (data S3). The expression patterns of these marker genes were visualized with either Seurat (*47*) or Scanpy (*49*).

### Cell cluster annotation and tissue data deconvolution

To integrate the single-cell and tissue-specific RNA-seq data, we performed a deconvolution analysis on the tissue-specific data using MuSiC (*21*). This method uses the gene expression information from single-cell RNA-seq data to estimate the cell composition of tissues based on their transcriptomic profiles.

To define marker genes for cell cluster identification in Aiptasia, we used previously identified markers from the soft coral *Xenia* (*23*). Orthologous gene groups between *Xenia* and Aiptasia were identified using OrthoFinder (*50*). The overlaps between cell markers in these two species were examined in R to assist with Aiptasia cell cluster annotation. To better understand the dominant function of each cell cluster, we performed a gene set variation analysis (GSVA) to determine the activities of annotated pathways in Aiptasia following a previously described workflow (*51*). The activities of the top five enriched pathways represented by GSVA scores were then visualized using ComplexHeatmap (*52*).

### Generating and verifying antibodies against glucose and ammonium transporters

To examine the tissue and cellular localizations of the major Aiptasia transporters for glucose (AipSLC2A8α, AIPGENE2706; AipGLUT1α, AIPGENE12082; AipGLUT8α, AIPGENE18406) and ammonium (AipAMT1, AIPGENE17420; AipRhBG1, AIPGENE18105), we generated rabbit polyclonal (AipSLC2A8α, AipGLUT1α, AipAMT1, and AipRhBG1; GenScript) or mouse monoclonal (AipGLUT8α; Abmart) antibodies against antigen peptides that are specific to each of the proteins (table S18).

To verify the specificities of the antibodies by Western blotting (fig. S6), Aiptasia cell lysates were prepared from ~20 anemones by homogenizing in ice-cold NP-40 cell lysis buffer (Thermo Fisher Scientific, #FNN0021) using TissueLyser II (QIAGEN). After determining the protein concentrations of freshly prepared homogenates using a DC protein assay kit (Bio-Rad), 20 μg (for AipSLC2A8α and AipGLUT1α), 15 μg (for AipGLUT8α), or 10 μg (for AipAMT1 and AipRhBG1) of the total protein per sample were then resolved on a 10% SDS–polyacrylamide gel electrophoresis gel and transferred onto a 0.45-μm polyvinylidene difluoride membrane using a wet/tank blotting system (Bio-Rad). The membrane was then blocked in 5% nonfat milk for 2 hours at ~23°C while samples of the antibodies were incubated with either TBST [20 mM tris, 150 mM NaCl (pH 7.6), and 0.1% Tween 20] or TBST supplemented with the appropriate antigen peptide (400 μM) for 2 hours at 4°C. The blocked membrane was then treated with the preincubated antibody (1:1000 in 5% nonfat milk) at 4°C overnight and then incubated with horseradish peroxidase (HRP)–conjugated goat anti-mouse immunoglobulin G (IgG) (1:20,000; for anti-AipGLUT8α) or HRP-conjugated goat anti-rabbit IgG secondary antibody (1:5000; for the other primary antibodies) for 1 hour at ~23°C. The immunoblots were then visualized by chemiluminescence using the ECL Western blotting substrate kit (Bio-Rad) and ChemiDoc XRS+ system (Bio-Rad). For antibodies still showing a weaker band with antigen preincubation, further blotting assays were performed with gradually increased concentrations of antigen peptide. The antibodies that were fully blocked by their appropriate antigen peptides were verified as antigen-specific. The Western blot–verified antibodies were then used for immunofluorescence staining experiments.

### Immunofluorescence staining of glucose and ammonium transporters

Anemones (symbiotic, aposymbiotic, or aposymbiotic treated with glucose) were relaxed in autoclaved seawater containing 3.75% (w/v) MgCl_2_ for ~15 min and then fixed overnight in freshly prepared 4% paraformaldehyde at 4°C. The fixed animals were dehydrated with a series of washes: PBS, 5 min; 50% ethanol, 5 min, twice; 70% ethanol, 5 min, twice; 80% ethanol, 5 min, twice; 90% ethanol, 5 min, twice; 100% ethanol, 10 min, twice; and xylene, 15 min, twice. The dehydrated specimens were then embedded in paraffin and cut into 5- or 10-μm sections (some of tentacles and some of body stalk) using a Leica RM2125 microtome. The sections were collected on glass slides and then dewaxed and rehydrated with seven steps of reversed washes: xylene; 100, 90, 80, 70, and 50% ethanol; and PBS containing 0.1% Triton X-100 to permeabilize the cells. After blocking at ~23°C for 30 min using a blocking buffer containing 5% normal goat serum, 1% bovine serum albumin (BSA), and 0.3% Triton X-100 (anti-AipSLC2A8α, anti-AipGLUT1α, anti-AipAMT1, and anti-AipRhBG1) or 5% normal goat serum, 1% BSA, and 0.1% Tween 20 (anti-AipGLUT8α), the sections were then incubated overnight at 4°C with the appropriate primary antibodies (for AipSLC2A8α, AipGLUT1α, AipAMT1, and AipRhBG1: 10 μg/ml in PBS containing 1% normal goat serum and 0.3% Triton X-100; for AipGLUT8α: a 1:500 dilution in the blocking buffer described above). Secondary antibodies [for AipSLC2A8α, AipGLUT1α, AipAMT1, and AipRhBG1: Alexa Fluor 555–conjugated goat anti-rabbit IgG (ab150078, Abcam) at 4 μg/ml in PBS containing 1% normal goat serum and 0.3% Triton X-100; for AipGLUT8α: a 1:1000 dilution of Alexa Fluor 488–conjugated goat anti-mouse IgG (ab150113, Abcam) in the blocking buffer described above] were then applied to the sections for 1 hour at ~23°C. The slides were washed briefly three times with PBS, stained for 2 min with Hoechst 33342 (5 μg/ml; H3570, Thermo Fisher Scientific), mounted with a ProLong Diamond antifade mountant (P36961, Thermo Fisher Scientific), and sealed with a clear nail polish. The same protocol was followed for negative controls where the tissue sections were stained with only secondary antibodies.

Fluorescence images showing tissue or cellular localizations of the transporters were then captured using the Leica Stellaris 8 FALCON (anti-AipSLC2A8α and anti-AipGLUT1α), a Keyence BZ-810 microscope (anti-AipGLUT8α), or a Leica TCS SP8 STED X (anti-AipAMT1 and anti-AipRhBG1). All images were then analyzed using Fiji (*53*).

### Rescue of yeast mutants with transformed Aiptasia transporters

To test the functions of symbiosis-induced putative glucose and ammonium transporters, we performed yeast mutant rescue experiments. For the glucose transporters, we amplified full-length transcripts for AipSLC2A8α, AipGLUT1α, and AipGLUT8α using gene-specific primers (table S20) and cloned the resulting cDNAs into the yeast vector pSH100 [YCplac33 MET25pro MCP-mCherry (*54*), a gift from R. Singer and D. Zenklusen (Addgene, plasmid no. 45930; http://n2t.net/addgene:45930; RRID:Addgene_45930)]. The constructs were then transformed into CFY07, a yeast mutant strain that has completely lost hexose uptake ability because of the concurrent knockout of 20 sugar transporter genes and an extracellular trehalase gene (*28*). For the ammonium transporters, we amplified full-length transcripts for AipAMT1 and AipRhGB1 using gene-specific primers (table S20) and cloned the resulting cDNAs into the yeast vector pYES2.1/V5-His-TOPO (K415001, Thermo Fisher Scientific). The resulting plasmids were sequenced to confirm sequence fidelity and transformed into the ammonium transporter–deficient mutant yeast strain 31019b, which lacks the three yeast ammonium transporter genes (*MEP1*, *MEP2*, and *MEP3*), at least one of which is essential for yeast to grow using ammonium as sole nitrogen source (*27*).

In all cases, yeast strains were grown overnight to exponential phase in complete medium [YPM (1% yeast extract, 2% peptone, and 2% maltose) for CFY07 and YPD (1% yeast extract, 2% peptone, and 2% glucose) for 31019b], pelleted from 1 ml of each culture, washed with Milli-Q water, and resuspended to a concentration of 1 × 10^4^ cells/μl. To test growth, dilution series were then prepared and plated on the appropriate synthetic medium, resulting in five colonies for each strain containing approximately 50,000, 5000, 500, 50, and 5 cells initially. CFY07 and its transformants were plated on a yeast nitrogen base (YNB) medium containing 2% glucose, whereas 31019b and its transformants were plated on Difco YNB medium without amino acids and ammonium (YNB-N) containing 20 mM NH_4_Cl and 2% galactose. Images showing cell growth were acquired after incubation at 30°C for 2 days.

To characterize the directionality of the ammonium transporters, the same 31019b transformants were plated on YNB-N medium containing 2% galactose, 200 mM methylammonium, and 0.1% proline. Proline can serve as the nitrogen source to support cell growth, but methylammonium is a toxic ammonium analog. Hence, cells expressing a unidirectional ammonium transporter accumulate the lethal chemical over time and die, whereas a bidirectional transporter can remove toxic methylammonium from the cells and thus allow them to survive. Images showing cell growth on the methylammonium plates were acquired 3 days after inoculation.

### Isotope labeling and NanoSIMS analysis

To investigate the dynamics of carbon and nitrogen assimilation in symbiotic and aposymbiotic animals, we performed stable isotope labeling experiments on two anemones of each type. Individual animals were incubated for 24 hours in 25-ml incubation chambers containing artificial seawater (ASW) at 25°C under a 12-hour light:12-hour dark cycle with a light intensity of 40 μmol photons m^−2^ s^−1^. The ASW was freshly prepared and contained NaCl (20.8 g/liter), MgCl_2_ (4.4 g/liter), Na_2_SO_4_ (3.5 g/liter), CaCl_2_ (1 g/liter_)_, and KCl (0.59 g/liter) at pH 8.2. It was supplemented with NaH^13^CO_3_ [>98 atom % (at %) of ^13^C] and ^15^NH_4_Cl (>98 at % of ^15^N) at final concentrations of 2 mM and 5 μM, respectively. Following the 24-hour incubations, all specimens were immediately transferred to a fixative solution [1.25% glutaraldehyde plus 0.5% paraformaldehyde in 0.1 M sodium phosphate buffer (pH 7.5)] and stored at 4°C for 24 hours before further processing.

Several individual tentacles were then collected from each animal using a stereomicroscope, postfixed for 1 hour at ~23°C in 1% OsO_4_ in 0.1 M sodium phosphate buffer (pH 7.5), and dehydrated using a series of rinses with increasing ethanol concentrations (50, 70, 90, and 100%) followed by 100% acetone. Tissues were then gradually infiltrated using Spurr’s resin (Electron Microscopy Sciences) at increasing concentrations (25, 50, and 75% in ethanol and then 100%) and finally embedded in 100% resin. Semithin sections (150 nm) were cut using a Leica Ultracut E microtome, mounted on silicon wafers (ProsciTech), and gold-coated using a Quorum Q150T sputter coater.

The gold-coated sections were then imaged using the NanoSIMS 50 ion probe at the Centre for Microscopy, Characterisation, and Analysis at the University of Western Australia. The surfaces of the samples were bombarded with a 16-keV primary Cs^+^ beam focused to a spot size of about 100 nm, with a current of ∼2 pA. Secondary molecular ions ^12^C^12^C^−^, ^12^C^13^C^−^, ^12^C^14^N^−^, and ^12^C^15^N^−^ were collected simultaneously in electron multipliers at a mass resolution (*M*/Δ*M*) > 8000, which is sufficient to resolve isobaric species (e.g., ^12^C^13^C^−^ from ^12^C^12^C^1^H^−^ and ^13^C^14^N^−^ from ^12^C^15^N^−^). Large mosaics of the different cell layers comprising, on average, 16 ± 3 images (45 μm raster with 512 by 512 pixels) were recorded for all targeted secondary ions by rastering the primary beam across the sample with a dwell time of 10 to 20 ms per pixel. After drift correction, the ^13^C/^12^C and ^15^N/^14^N maps were generated based on ^12^C^13^C^−^/^12^C^12^C^−^ and ^12^C^15^N^−^/^12^C^14^N^−^ ratios as hue-saturation-intensity images, where the color scale represents the isotope ratio, and further analyzed using Fiji with the Open-MIMS plug-in (https://github.com/BWHCNI/OpenMIMS/wiki). Isotopic enrichments in the Aiptasia mosaics were quantified in the epidermis, gastrodermis (excluding the algal cells in symbiotic samples; fig. S10), and algal cells by extracting data from each pixel (spatial resolution of ~88 nm) in the regions of interest across the tentacle cross sections. More than 10^6^ pixel values were extracted for each tissue layer. To eliminate potential effects on the subsequent statistical analyses that might be caused by different sample sizes (total pixel numbers), the pixel values from each tissue layer were randomly assigned into 10,000 bins. The ^13^C/^12^C and ^15^N/^14^N ratios were then used to quantify the isotope enrichment for each bin. The Pearson correlations between ^13^C and ^15^N enrichments were calculated based on the relative signal intensities for each treatment and tissue layer, and the isotope enrichment levels from different tissues were compared using the R package cocor (*55*). In addition, the same NanoSIMS mosaics were used to approximate the relative overall contributions by the anemone tissues and the algae to ammonium assimilation in the holobiont. For this, the total ^15^N counts across the regions of interest were corrected for the background levels of natural isotope abundance (based on isotope ratios from yeast and unlabeled Aiptasia samples) to quantify the excess ^15^N assimilation by each symbiotic partner.

## Notes

View/request a protocol for this paper from *Bio-protocol*.
