# Supplementary material for: Molecular insights into the Darwin paradox of coral reefs from the sea anemone Aiptasia
Source: Sci Adv. 2023 Mar 15;9(11):eadf7108. doi: 10.1126/sciadv.adf7108 (PMC10017044; doi:10.1126/sciadv.adf7108)
Supplement: Supplementary file 1 — Fig. S1 to S10 Table S1 to S20 Legends for data S1 to S5 [file sciadv.adf7108_sm.pdf]

Science Advances
AAAS

Supplementary Materials for

Molecular insights into the Darwin paradox of coral reefs from the sea anemone Aiptasia

Guoxin Cui *et al.*

Corresponding author: Guoxin Cui, guoxin.cui@kaust.edu.sa; Manuel Aranda, manuel.aranda@kaust.edu.sa

Sci. Adv. **9**, eadf7108 (2023)
DOI: 10.1126/sciadv.adf7108

The PDF file includes:

Fig. S1 to S10
Table S1 to S20
Legends for data S1 to S5

Other Supplementary Material for this manuscript includes the following:

Data S1 to S5

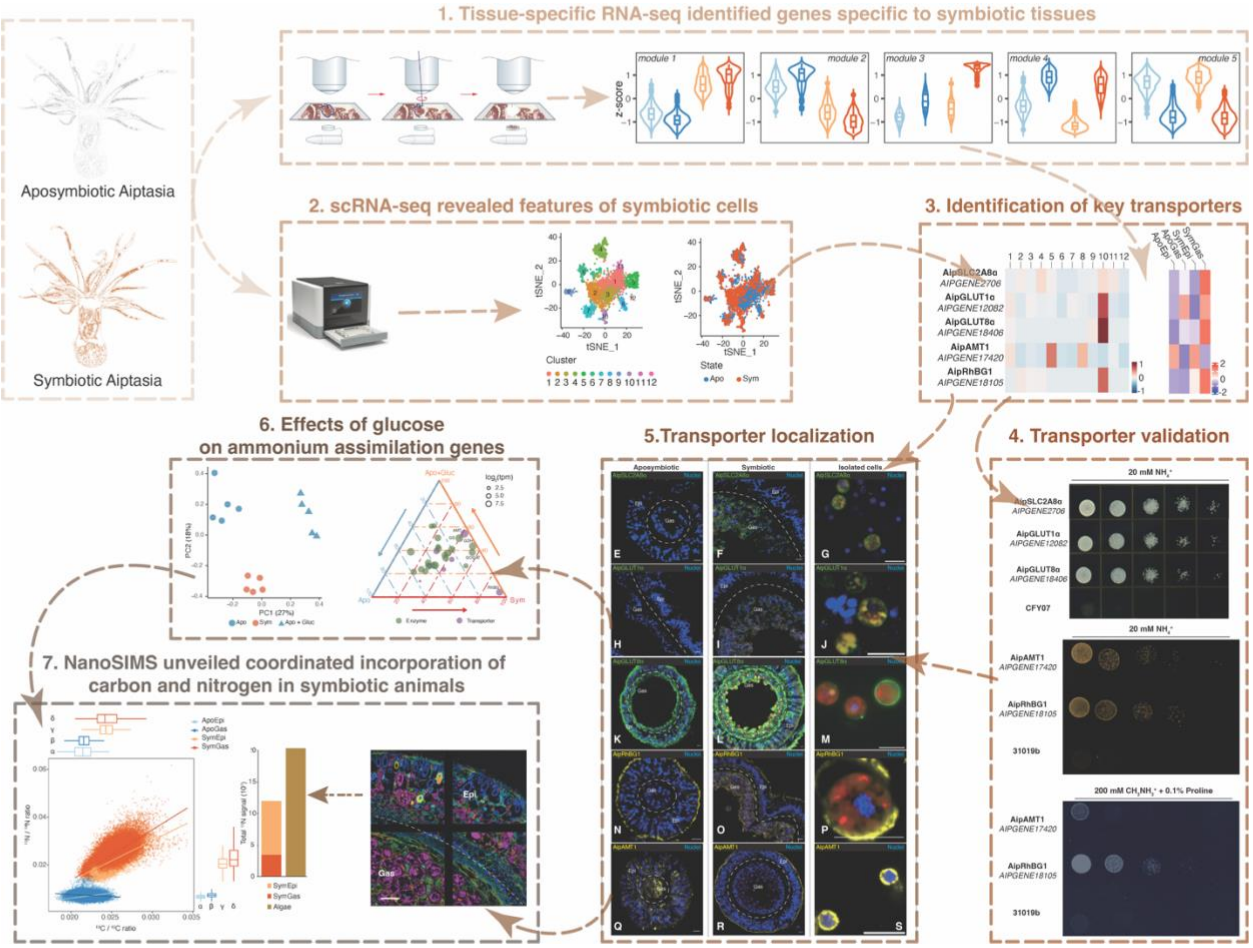

Fig. S1. Summary of the study. Using aposymbiotic and symbiotic anemones, we performed laser-microdissection-assisted tissue-specific transcriptomic profiling and identified gene modules associated with the symbiotic tissue layer (**1**). To further examine symbiosis-induced gene expression changes at the single-cell level, we conducted single-cell RNA-seq (**2**). Combined tissue-specific and scRNA-seq data highlighted five key symbiosis-associated nutrient transporters (**3**). Using yeast mutant rescue experiments, we verified and characterized the function of these transporters (**4**). Immunofluorescence staining showed that the expression and localization of these transporters are altered by symbiosis (**5**). Relocalization and expression changes were joint effects of increased glucose availability and the presence of the symbiont (**6**). The combined data suggested a promoted co-incorporation of carbon and nitrogen in symbiotic animals, which was confirmed through NanoSIMS analysis of ^{13}C and ^{15}N labeling experiments (**7**).

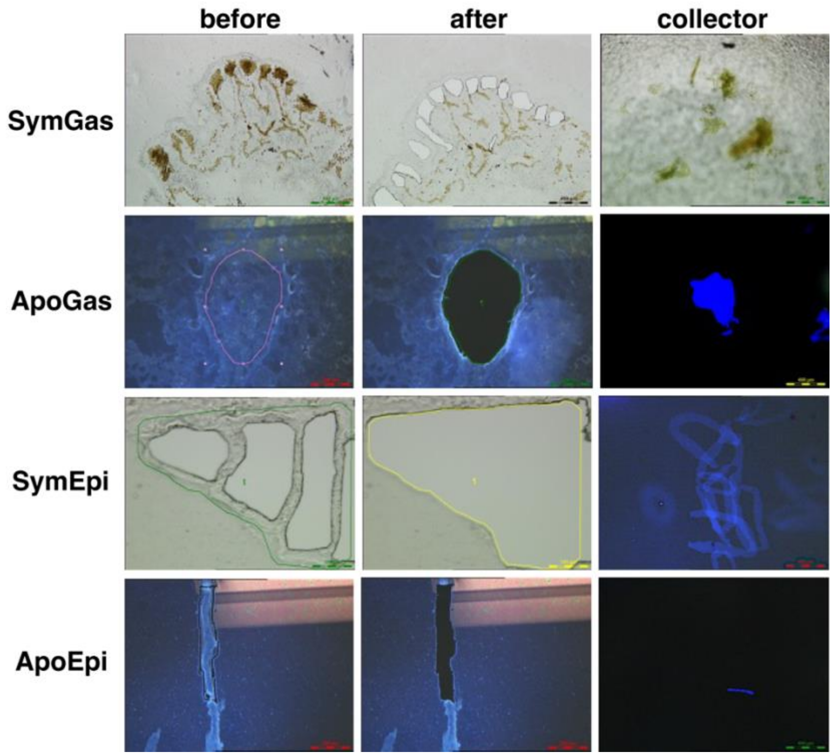

Fig. S2. Laser microdissection (LMD) of symbiotic and aposymbiotic Aiptasia. Symbiotic samples were imaged under white light, whereas aposymbiotic samples were visualized using a Leica filter cube B/G/R or A, which helped in identifying their epidermal cell layer. Sym, symbiotic; Apo, aposymbiotic; Gas, gastrodermis; Epi, epidermis. Brightness and contrast were adjusted for the collector images to increase the visibility of the tissues collected.

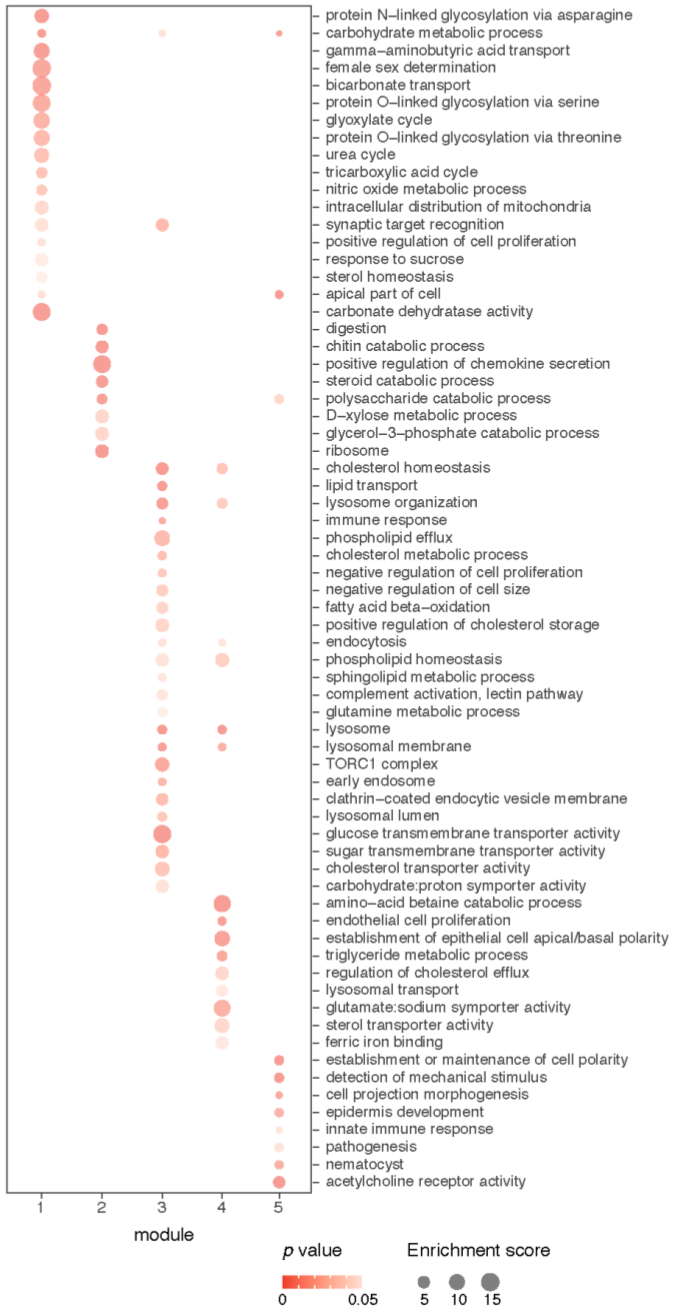

Fig. S3. Gene-ontology (GO) terms enriched by the genes in each of the five modules identified from multi-factorial differential-expression analysis (Fig. 1C). The size of each dot represents the enrichment score that was calculated by dividing the actual by the expected number of DEGs associated with the corresponding term.

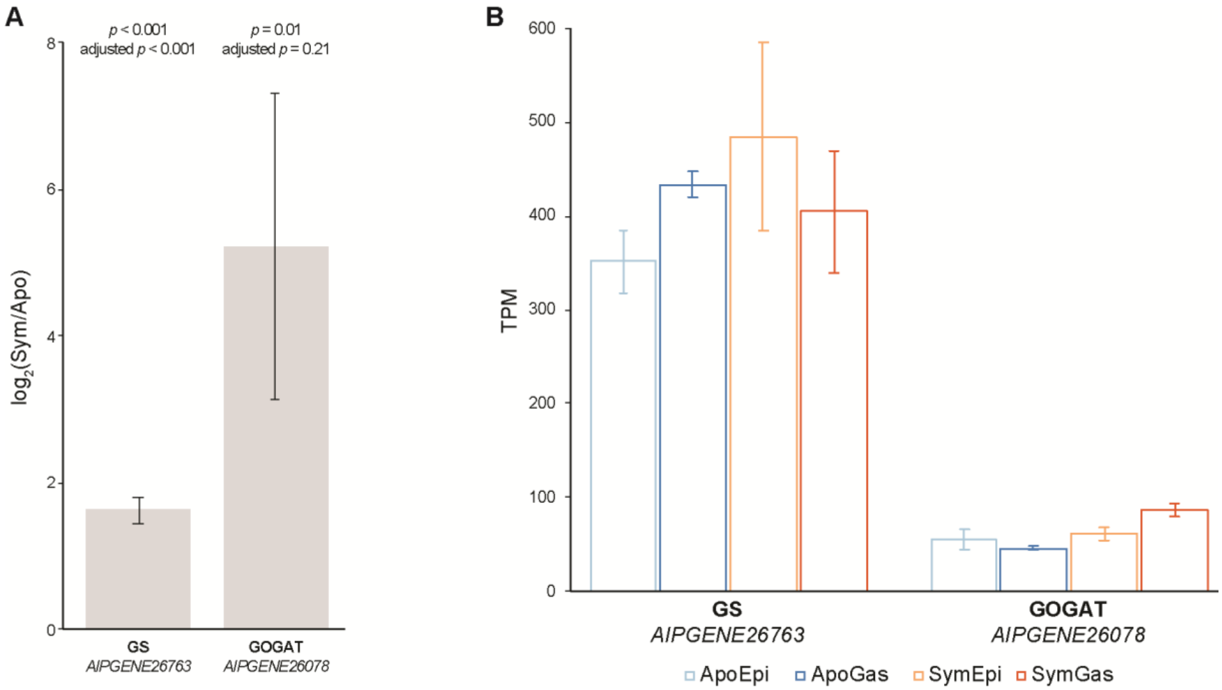

Fig. S4. Expression patterns of key genes associated with ammonium assimilation at organism (A) and tissue (B) levels. Organism-level data in (**A**) were extracted from Cui et al. (15). GS, glutamine synthetase; GOGAT, glutamate synthase. Error bars represent mean ± SE.

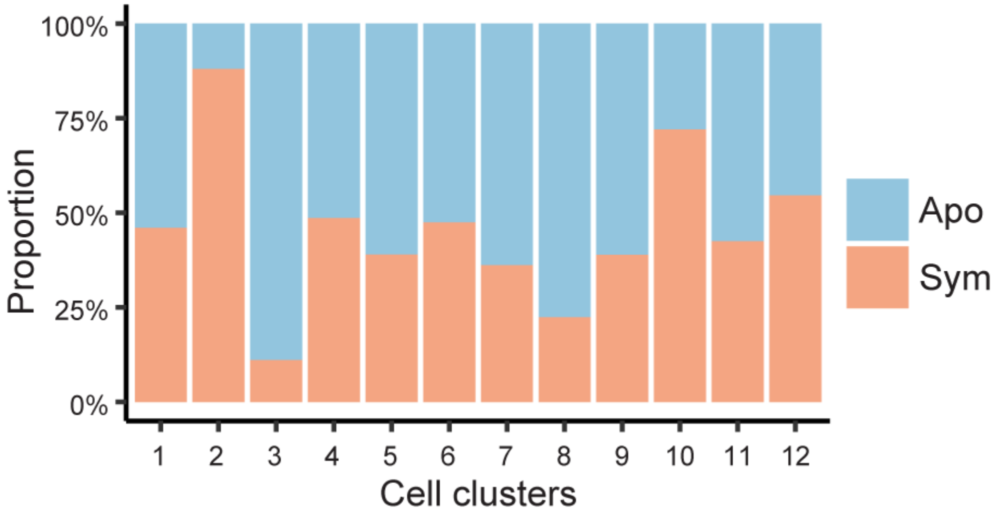

Fig. S5. Proportions of cells originating from aposymbiotic or symbiotic anemones in the 12 clusters identified by single-cell RNA-Seq (see **Fig. 1, D and E**). Apo, aposymbiotic; Sym, symbiotic.

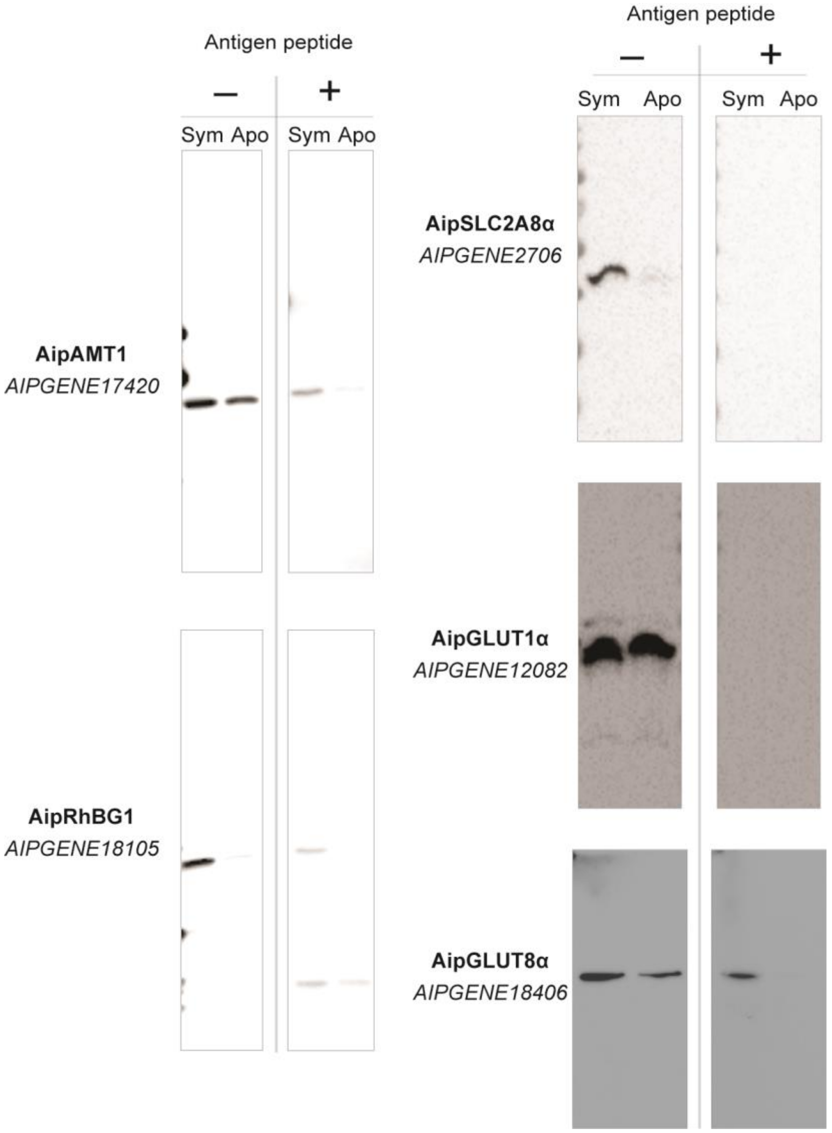

Fig. S6. Western-blot validation of the custom antibodies. 10 μg (for AipAMT1 and AipRhBG1), 15 μg (for AipGLUT8α), or 20 μg (for AipSLC2A8α and AipGLUT1α) of total protein isolated from symbiotic (Sym) or aposymbiotic (Apo) anemones were resolved on SDS-PAGE gels and transferred onto PVDF membranes. The membranes were incubated either with only the appropriate antibody (-) or with the appropriate antibody pre-absorbed with its antigen peptide (+). Further details are in Materials and Methods.

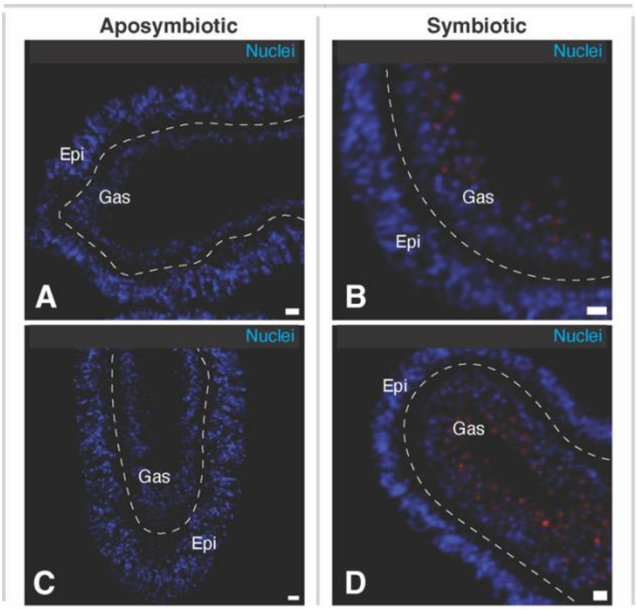

Fig. S7. Immunofluorescence staining of aposymbiotic (A, C) and symbiotic (B, D) Aiptasia with only the secondary antibodies: Alexa Fluor 488-conjugated goat anti-mouse-IgG (**A, B**); Alexa Fluor 555-conjugated goat anti-rabbit-IgG (**C, D**). The images shown are representative of many such cells observed. Scale bars (all panels), 10 µm.

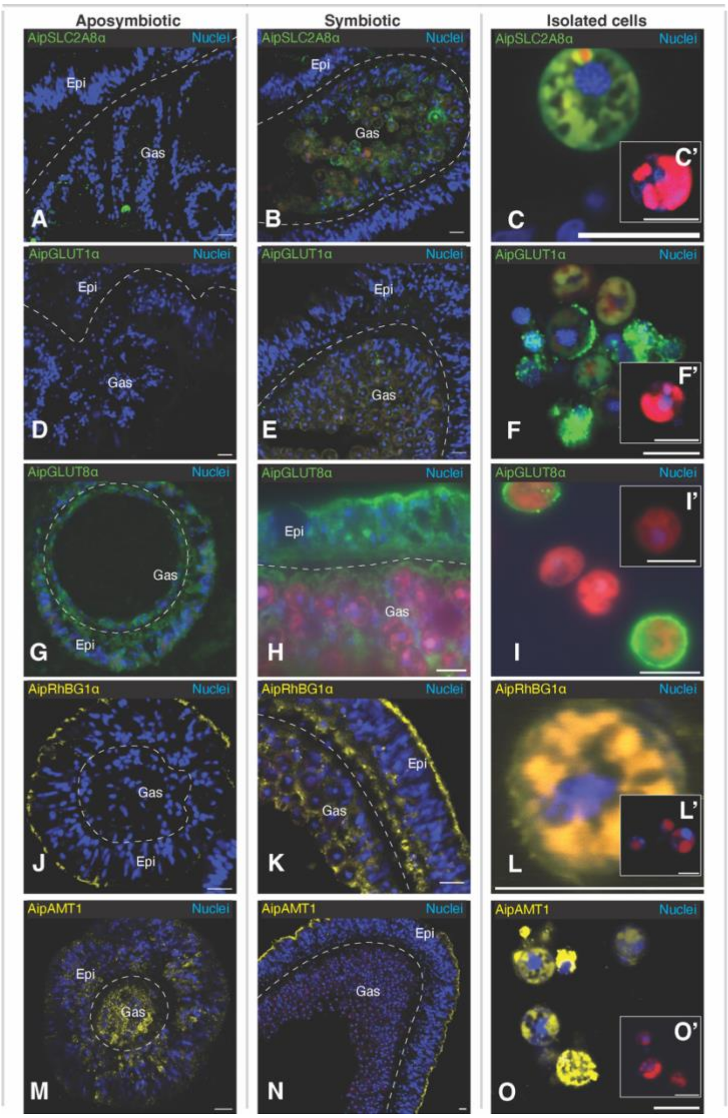

Fig. S8. Additional immunofluorescence images (as in Fig. 2, E – S) showing relocalization of glucose (A – I) and ammonium (J – O) transporters induced by symbiosis. Tissue sections of aposymbiotic (**A, D, G, J, M**) and symbiotic (**B, E, H, K, N**) anemones are shown along with cells isolated from symbiotic animals (**C, F, I, L, O**) and cultured algal cells (strain SSB01; **C', F', I', L', O'**). The image shown is representative of many such tissue sections and cells observed. Scale bars (all panels), 10 μm.

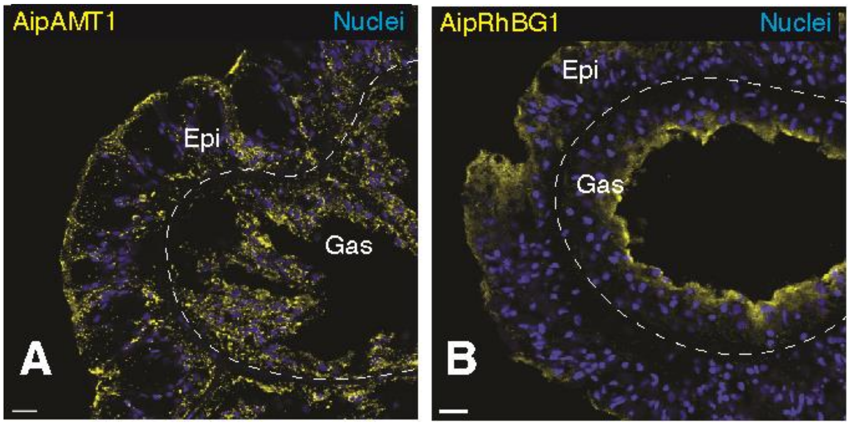

Fig. S9. Additional images showing localization patterns of ammonium transporters AipAMT1 (A) and AipRhGB1 (B) in glucose-treated aposymbiotic Aiptasia (see Fig. 3 C and D). Scale bars (both panels), 10 μm.

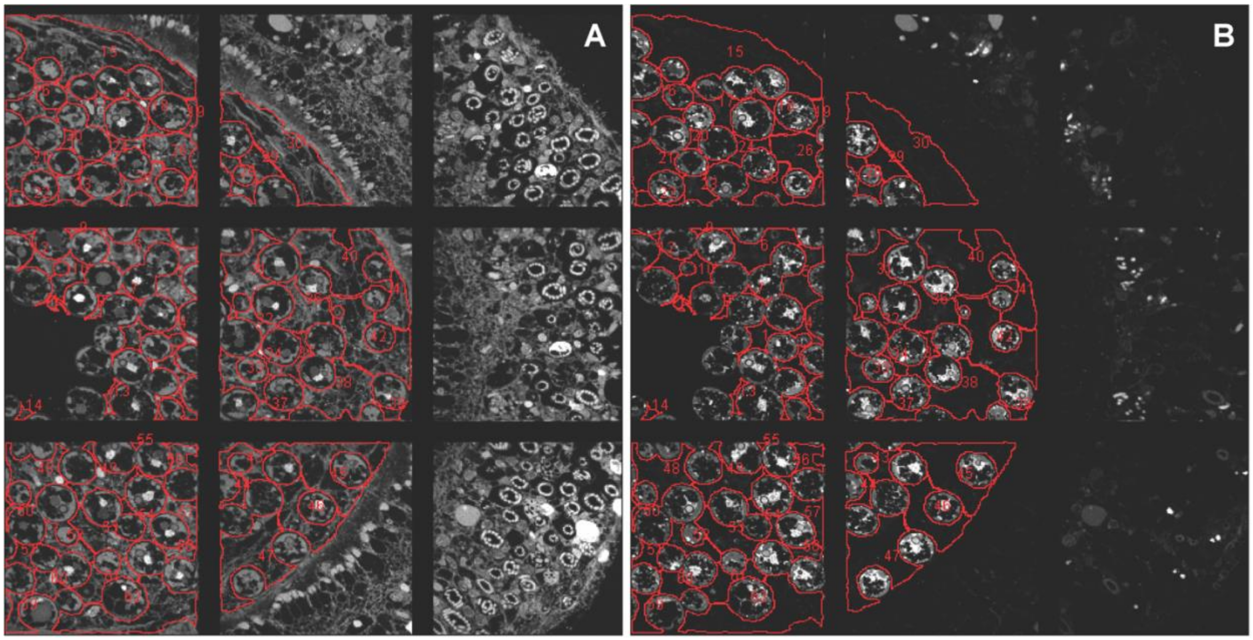

Fig. S10. Representative regions of interest (ROI; numbered red outlines) in the gastrodermis displayed for (A) ^{14}N and (B) ^{15}N. The ROI were drawn manually to include only gastrodermal tissue and not algal-symbiont cells.

Table S1 Module 1 genes involved in carbon cycling and carbohydrate metabolism

Gene ID[a,b]	Average expression level (TPM)[c]		Fold change[c]		Annotation
	SymEpi	SymGas	SymEpi/ApoEpi	SymGas/ApoGas	
AIPGENE17854	51	110	2.4	6.0	Isocitrate lyase
AIPGENE21331	85	220	1.3	5.7	Carnitine O-palmitoyltransferase 1
AIPGENE15469	39	53	1.5	5.4	Carbonic anhydrase 7
AIPGENE6376	59	76	3.4	4.5	Phosphorylase b kinase gamma catalytic chain PHKG2
AIPGENE2308	40	56	1.6	4.2	Polypeptide N-acetylgalactosaminyltransferase 1
AIPGENE12054	26	31	1.9	3.7	Glucose transporter type 1
AIPGENE12939	15	26	1.6	3.7	Alpha-N-acetylgalactosaminidase NAGA
AIPGENE9860	50	53	2.8	3.6	[Pyruvate dehydrogenase (acetyl-transferring)] kinase isozyme 2 PDK2
AIPGENE8506	25	34	2.0	3.0	Solute carrier family 26 member 6 SLC26A6
AIPGENE21162	19	24	1.6	2.9	Carbohydrate sulfotransferase 1
AIPGENE1116	19	30	1.8	2.7	N-acetylglucosamine-6-phosphate deacetylase amdhd2
AIPGENE17843	50	64	2.6	2.6	Malate synthase
AIPGENE24851	70	82	1.4	2.4	Serine/threonine-protein kinase/endoribonuclease IRE1A
AIPGENE12778	49	68	1.3	2.2	Galactokinase GALK1
AIPGENE5850	53	50	1.1	1.9	Capsule biosynthesis protein CapA
AIPGENE6132	20	22	2.1	1.8	Lysosomal alpha-mannosidase
AIPGENE3057	62	69	1.8	1.7	Pyruvate carboxylase
AIPGENE22466	27	32	1.5	1.7	UDP-glucose:glycoprotein glucosyltransferase 1
AIPGENE18740	36	41	1.6	1.6	Neutral alpha-glucosidase AB
AIPGENE25712	34	18	4.1	1.4	Di-N-acetylchitobiase CTBS
AIPGENE28157	110	62	2.1	1.3	Carbamoyl-phosphate synthase [ammonia] CPS1

[a] Ordered by SymGas/ApoGas.

[b] Gray highlight, a gene encoding a putative glucose transporter that is upregulated in both gastrodermal and epidermal cells of symbiotic animals but not investigated further in this study; yellow highlight, genes encoding glyoxylate-cycle enzymes.

[c] Numbers are rounded to two significant digits.

Table S2 Module 1 genes involved in nitrogen cycling and serine/glycine biosynthesis

Gene ID[a,b]	Average expression level (TPM)[c]		Fold change[c]		Annotation
	SymEpi	SymGas	SymEpi/ApoEpi	SymGas/ApoGas	
AIPGENE18105	20	410	6.8	170	Ammonium transporter Rh type B
AIPGENE18835	26	28	2.0	3.7	Glycine N-methyltransferase GNMT
AIPGENE22717	48	72	2.4	3.1	Serine-pyruvate aminotransferase AGXT
AIPGENE18470	27	23	1.7	1.8	Argininosuccinate synthase
AIPGENE28157	110	62	2.1	1.3	Carbamoyl-phosphate synthase [ammonia] CPS1

[a] Ordered by SymGas/ApoGas.

[b] Gray highlight, a gene encoding an ammonium transporter (AipRhBG1) that is investigated further in this study.

[c] Numbers are rounded to two significant digits.

Table S3 Module 1 genes involved in post-translational modification

Gene ID[a,b]	Average expression level (TPM)[c]		Fold change[c]		Annotation
	**SymEpi**	**SymGas**	**SymEpi/ApoEpi**	**SymGas/ApoGas**	
AIPGENE130	45	100	2.4	4.6	Alpha-1,3-Glucosyltransferase ALG8
AIPGENE16395	28	26	1.5	3.8	Low-salt glycan biosynthesis protein Agl12
AIPGENE8406	29	17	2.0	2.5	Polypeptide N-acetylgalactosaminyltransferase 1 GALNT1
AIPGENE26605	16	16	2.6	2.3	GPI ethanolamine phosphate transferase 3 PIGO
AIPGENE15461	18	18	1.8	1.9	Dolichyl-phosphate beta-glucosyltransferase ALG5
AIPGENE4856	110	90	3.2	1.8	Protein transport protein Sec24C
AIPGENE6178	48	49	1.9	1.8	Dolichyl-diphosphooligosaccharide--protein glycosyltransferase STT3B
AIPGENE2497	56	53	1.9	1.6	Protein transport protein Sec23A
AIPGENE27679	93	97	1.5	1.2	Protein transport protein Sec24B
AIPGENE16	60	21	3.4	1.1	Alpha-1,3-glucosyltransferase ALG6

[a] Ordered by SymGas/ApoGas.

[b] Genes potentially involved in N-linked glycosylation are highlighted.

[c] Numbers are rounded to two significant digits.

Table S4 Module 2 genes involved in food digestion

Gene ID[a,b]	Average expression level (TPM)[c]		Fold change[c]		Annotation
	ApoEpi	ApoGas	ApoEpi/SymEpi	ApoGas/SymGas	
AIPGENE9704	6.8	35	8.2	58	Chymotrypsinogen B
AIPGENE25876	290	1500	32	55	Chymotrypsin B
AIPGENE15455	98	400	12	51	Chitinase 3
AIPGENE2028	35	150	8.1	48	Trypsin-3
AIPGENE8235	71	380	15	35	Anionic trypsin
AIPGENE23293	200	740	13	27	Acidic mammalian chitinase
AIPGENE24288	59	210	2.6	21	Chymotrypsin-like protease CTRL-1
AIPGENE1364	77	180	3.9	19	Meprin A subunit beta MEP1B
AIPGENE2734	22	93	11	17	Chymotrypsinogen B
AIPGENE28200	30	85	8.1	16	Acidic mammalian chitinase
AIPGENE2019	10	36	4.3	15	Trypsin-3
AIPGENE23157	9.3	27	3.3	13	Chymotrypsinogen B
AIPGENE28199	14	22	5.2	12	Acidic mammalian chitinase
AIPGENE7785	24	74	6.8	9.8	Chitinase 3
AIPGENE17423	8.7	24	3.1	9.6	Maltase-glucoamylase MGAM
AIPGENE26805	10	35	3.2	9.1	Chymotrypsinogen B
AIPGENE24686	17	18	1.0	3.1	Beta-hexosaminidase HEX
AIPGENE6698	52	63	1.7	2.9	Chymotrypsinogen B2

[a] Ordered by ApoGas/SymGas.

[b] Genes encoding putative chitinases are highlighted; they should allow digestion of a major exoskeletal component of most cnidarian prey animals.

[c] Numbers are rounded to two significant digits.

Table S5 Module 3 genes involved in lysosomal/symbiosomal organization

Gene ID[a,b]	Average expression level (TPM)[c]		Fold change[c]		Annotation
	ApoGas	SymGas	SymGas/SymEpi	SymGas/ApoGas	
AIPGENE18610	17	840	18	50	Sulfated glycoprotein 1
AIPGENE20198	1.3	42	13	32	Sulfotransferase 1C2A
AIPGENE762	10	160	8.0	15	Phospholipase B-like 2
AIPGENE8500	14	110	10	8.3	Gamma-glutamyl hydrolase
AIPGENE756	12	90	5.3	7.5	Phospholipase B-like 2
AIPGENE8201	19	120	5.2	6.6	Gamma-glutamyl hydrolase
AIPGENE9365	19	120	4.3	6.4	Beta-glucuronidase
AIPGENE9310	78	490	7.2	6.3	E3 ubiquitin-protein ligase MARCH1
AIPGENE17332	42	230	3.2	5.7	Regulatory-associated protein of mTOR
AIPGENE12986	14	78	4.1	5.6	Bcl-2-like protein 1
AIPGENE2358	21	110	8.2	5.3	Deleted in malignant brain tumors 1 protein DMBT1
AIPGENE17384	34	170	4.4	4.9	Regulatory-associated protein of mTOR
AIPGENE1615	9.3	45	5.1	4.9	Vacuolar protein sorting-associated protein 4B
AIPGENE13031	11	51	3.3	4.5	Alpha-N-acetylgalactosaminidase
AIPGENE9338	25	93	3.8	3.8	Beta-glucuronidase
AIPGENE23106	45	170	3.0	3.7	Legumain
AIPGENE10665	33	100	5.9	3.1	Sulfotransferase 1C2A
AIPGENE8377	29	84	2.7	2.9	Sorting nexin-13
AIPGENE11822	33	89	3.3	2.7	AP-5
AIPGENE16722	26	62	2.0	2.4	Lysosomal thioesterase PPT2-A
AIPGENE1266	25	59	2.8	2.4	Somatomedin-B and thrombospondin type-1 domain-containing protein
AIPGENE14910	31	66	9.1	2.1	Basement membrane-specific heparan sulfate proteoglycan core protein HSPG2
AIPGENE25198	75	149	1.8	2.0	PTB domain-containing engulfment adapter protein 1
AIPGENE13027	46	87	2.3	1.9	Alpha-N-acetylgalactosaminidase

AIPGENE23952	6	113	3.2	1.8	Nischarin
AIPGENE27709	32	60	1.5	1.8	Lysosome membrane protein 2 SCARB2
AIPGENE5637	28	50	2.5	1.8	Phospholipase B-like 1
AIPGENE1792	18	31	2.1	1.7	Chitinase domain-containing protein 1
AIPGENE17107	25	41	1.7	1.6	Vacuolar protein sorting-associated protein 33A
AIPGENE1844	43	69	3.1	1.6	Steryl-sulfatase
AIPGENE20604	27	42	1.7	1.6	H(+)/Cl(-) exchange transporter 7

[a] Ordered by SymGas/ApoGas.

[b] Some genes encoding lysosomal marker proteins are highlighted.

[c] Numbers are rounded to two significant digits.

Table S6 Module 3 genes involved in sugar transport

Gene ID[a,b]	Average expression level (TPM)[c]		Fold change[c]		Annotation
	SymEpi	SymGas	SymGas/SymEpi	SymGas/ApoGas	
AIPGENE18406	10	70	7.8	6.9	Facilitated glucose transporter GLUT8
AIPGENE27196	23	140	9.9	6.2	Solute carrier family 23 member 2 SLC23A2
AIPGENE2706	5.0	21	9.5	4.7	Solute carrier family 2, facilitated glucose transporter member 8 SLC2A8
AIPGENE6170	8.0	19	2.0	2.5	Solute carrier family 2, facilitated glucose transporter member 8 SLC2A8
AIPGENE12097	23	49	1.6	2.2	Solute carrier family 2, facilitated glucose transporter member 1 SLC2A1

[a] Ordered by SymGas/ApoGas.

[b] Gray highlight, genes encoding glucose transporters that are investigated further in this study; yellow highlight, genes encoding putative glucose transporters that are upregulated in symbiotic gastrodermal cells but not investigated further in this study.

[c] Numbers are rounded to two significant digits.

Table S7 Module 3 genes involved in sterol transport and homeostasis

Gene ID[a,b]	Average expression level (TPM)[c]		Fold change[c]		Annotation
	ApoGas	SymGas	SymGas/SymEpi	SymGas/ApoGas	
AIPGENE23673	3.7	70	15	19	Lathosterol oxidase
AIPGENE2187	24	390	9.9	16	Low-density lipoprotein receptor-related protein 6
AIPGENE3074	46	460	13	10	Protein NPC2
AIPGENE15716	45	330	4.9	7.3	Prolow-density lipoprotein receptor-related protein 1
AIPGENE13786	71	450	4.4	6.3	Lipid storage droplets surface-binding protein 2
AIPGENE12169	8.6	49	4.0	5.7	Low density lipoprotein receptor adapter protein 1
AIPGENE23723	850	4000	8.5	4.7	Apolipophorins
AIPGENE20806	23	60	2.2	2.6	Hormone-sensitive lipase
AIPGENE7350	22	44	4.3	2.0	Long-chain-fatty-acid--CoA ligase 5
AIPGENE23362	190	280	1.9	1.5	Sterol regulatory element-binding protein 1
AIPGENE23053	31	34	1.4	1.1	Non-specific lipid-transfer protein SCP2

[a] Ordered by SymGas/ApoGas.

[b] A cholesterol-transport protein previously characterized in Aiptasia and other cnidarians is highlighted.

[c] Numbers are rounded to two significant digits.

Table S8 Module 4 genes involved in digestion

Gene ID[a,b]	Average expression level (TPM)[c]		Fold change[c]		Annotation
	ApoGas	SymGas	ApoGas/ApoEpi	SymGas/SymEpi	
AIPGENE21470	190	380	2.4	6.7	Proactivator polypeptide
AIPGENE18552	92	89	2.1	6.4	Acetylcholinesterase
AIPGENE27509	65	99	1.8	4.6	Betaine--homocysteine S-methyltransferase 1
AIPGENE26157	680	350	2.8	4.1	Cathepsin L
AIPGENE16849	170	180	2.1	3.5	Cathepsin B
AIPGENE10977	23	21	2.2	3.3	Betaine--homocysteine S-methyltransferase 1
AIPGENE203	20	21	2.1	3.2	Tripeptidyl-peptidase 1
AIPGENE4319	21	9.3	2.2	3.0	Arylsulfatase B
AIPGENE27525	40	43	1.6	3.0	Betaine--homocysteine S-methyltransferase 1
AIPGENE20697	40	56	1.0	2.9	Betaine--homocysteine S-methyltransferase 1
AIPGENE25614	10	11	2.1	2.6	Lysosome membrane protein 2
AIPGENE7709	29	18	2.8	2.4	E3 ubiquitin-protein ligase MIB1
AIPGENE14803	28	15	2.7	2.3	E3 ubiquitin-protein ligase MIB1
AIPGENE462	11	8.5	1.2	2.2	Chymotrypsinogen B
AIPGENE28006	30	22	2.2	2.0	Alpha-N-acetylglucosaminidase
AIPGENE19389	29	25	2.1	1.8	N-acylethanolamine-hydrolyzing acid amidase
AIPGENE28710	41	39	2.4	1.7	Arylsulfatase B
AIPGENE20962	110	89	1.5	1.5	Lysosomal-trafficking regulator
AIPGENE28230	24	5.4	3.7	1.3	Sulfotransferase 1C2A

[a] Ordered by SymGas/SymEpi.

[b] Genes potentially involved in protein degradation are highlighted.

[c] Numbers are rounded to two significant digits.

Table S9 Module 5 genes involved in cnidocyte function

Gene ID[a,b]	Average expression level (TPM)[c]		Fold change[c]		Annotation
	ApoEpi	SymEpi	ApoEpi/ApoGas	SymEpi/SymGas	
AIPGENE23882	10	9.0	5.7	90	Nematocyte expressed protein 6
AIPGENE21874	11	35	11	19	Equinatoxin-2
AIPGENE3089	15	39	3.8	15	Toxin PsTX-60B
AIPGENE8715	12	37	8.0	15	Toxin CfTX-2
AIPGENE5924	63	320	2.6	10	minicollagen 1
AIPGENE3166	19	44	6.3	9.8	Toxin AvTX-60A
AIPGENE11611	58	190	2.9	8.1	Nematocyst outer wall antigen
AIPGENE4696	45	69	37	8.1	Phospholipase A2
AIPGENE13265	46	76	2.9	6.3	Nematocyte expressed protein 6
AIPGENE17670	9.6	30	5.8	4.3	Toxin CaTX-A
AIPGENE3053	15	13	2.4	4.2	Toxin AvTX-60A
AIPGENE17675	8.5	47	2.2	3.7	Toxin CaTX-A
AIPGENE19223	6.3	6.8	2.8	3.4	Toxin CaTX-A

[a] Ordered by SymEpi/SymGas.

[b] Four previously well characterized nematocyte markers are highlighted; two of them are shown in Fig. 1G.

[c] Numbers are rounded to two significant digits.

Table S10 Module 5 genes involved in responses to mechanical stimuli

Gene ID[a,b]	Average expression level (TPM)[c]		Fold change[c]		Annotation
	ApoEpi	SymEpi	ApoEpi/ApoGas	SymEpi/SymGas	
AIPGENE6717	20	55	2.8	15	Amyloid beta A4
AIPGENE26233	54	67	3.2	7.5	Tetraspan membrane protein of hair cell stereocilia
AIPGENE7279	130	190	6.9	7.2	Nephrocystin-3
AIPGENE727	21	13	3.6	6.9	Acid-sensing ion channel 3
AIPGENE13869	48	300	3.0	6.2	YadA domain protein
AIPGENE16071	15	14	5.2	5.2	Acid-sensing ion channel 3
AIPGENE7259	58	58	5.1	4.8	Nephrocystin-3
AIPGENE7280	31	43	3.5	4.7	Nephrocystin-3
AIPGENE7264	150	120	2.5	4.4	Nephrocystin-3
AIPGENE10398	44	40	4.7	4.1	Nephrocystin-3
AIPGENE18522	12	11	3.3	3.7	Acid-sensing ion channel 3
AIPGENE7455	7.1	6.5	3.2	3.6	Acid-sensing ion channel 3
AIPGENE565	44	12	6.6	3.2	Transient receptor potential cation channel subfamily A member 1 TRPA1
AIPGENE3114	20	28	5.5	3.2	Nephrocystin-3
AIPGENE3223	78	75	8.1	3.0	Nephrocystin-3
AIPGENE13728	29	26	5.8	2.7	Transient receptor potential cation channel subfamily A member 1 TRPA1
AIPGENE4254	43	54	2.5	2.3	Nephrocystin-3
AIPGENE1377	34	32	1.2	2.0	Acid-sensing ion channel 3
AIPGENE15296	20	18	2.5	2.0	Nephrocystin-3
AIPGENE20487	350	270	1.9	1.3	Nephrocystin-3
AIPGENE20323	79	59	2.0	1.2	Nephrocystin-3

[a] Ordered by SymEpi/SymGas.

[b] Genes potentially required for normal ciliary development and function are highlighted.

[c] Numbers are rounded to two significant digits.

Table S11 Potential cnidocyte marker genes in Cluster 11

Gene ID[a,b]	Fold change[c]	Adjusted *p* value	Annotation
AIPGENE5924	37	5E-25	Minicollagen 1
AIPGENE23847	20	3E-18	Minicollagen 2
AIPGENE25615	14	7E-40	Venom prothrombin activator omicarin-C
AIPGENE11170	7.4	3E-12	Latrophilin-3
AIPGENE11611	6.1	9E-33	Nematocyst outer wall antigen
AIPGENE29070	5.5	1E-05	Matrilin-2
AIPGENE25580	5.0	9E-88	Lactadherin OS=Rattus norvegicus
AIPGENE2293	3.7	9E-88	D-galactoside-specific lectin
AIPGENE23596	2.0	6E-49	L-rhamnose-binding lectin CSL1

[a] Ordered by Fold change.

[b] Genes shown in Fig. 1G are highlighted.

[c] Fold-changes calculated by comparing the cells in Cluster 11 with all the other cells.

Table S12 Potential gastrodermal-cell marker genes in Cluster 2, 7, and/or 10

Cluster	Gene ID[a,b]	Fold change[c]	Adjusted *p* value	Annotation
2	AIPGENE21625	2.5	1E-18	Titin
2	AIPGENE3988	2.0	4E-05	Tropomyosin
7	AIPGENE6165	8.2	5E-22	Integrin alpha-8
7	AIPGENE23790	6.0	3E-60	Integrin beta-6
7	AIPGENE27486	5.5	4E-21	Tropomyosin alpha-4
7	AIPGENE23262	4.5	6E-56	Fibroblast growth factor receptor 1
7	AIPGENE5861	4.5	2E-18	Integrin beta-2
7	AIPGENE22443	3.7	1E-28	Soma ferritin
7	AIPGENE21255	3.7	2E-12	Integrin alpha-4
7	AIPGENE16019	3.0	4E-22	Fibroblast growth factor receptor 3
7	AIPGENE8440	2.7	1E-11	Integrin alpha-6
7	AIPGENE15963	2.5	1E-11	Laminin beta-1
7	AIPGENE17012	2.2	3E-12	Laminin alpha-4
7	AIPGENE6003	2.0	4E-07	Cathepsin L
7	AIPGENE26156	2.0	5E-17	Cathepsin L
7	AIPGENE2368	2.0	5E-06	Laminin gamma-1
10	AIPGENE3975	5.5	1E-25	Fibroblast growth factor receptor
10	AIPGENE22204	3.7	3E-23	Cathepsin B
10	AIPGENE23262	2.5	7E-11	Fibroblast growth factor receptor 1
10	AIPGENE26157	2.2	7E-13	Cathepsin L

[a] Ordered by Fold change within each cluster.

[b] Potential marker genes were selected according to cell markers identified in other cnidarian species.

[c] Fold-changes calculated by comparing the cells in Cluster 2, 7, or 10 with all the other cells.

Table S13 Cluster 10 marker genes associated with lysosomal organization and function

Gene ID[a]	Fold change[b]	Adjusted *p* value	Annotation
AIPGENE22204	3.7	3E-23	Cathepsin B
AIPGENE21470	3.3	3E-16	Proactivator polypeptide
AIPGENE5531	3.3	3E-21	Niemann-Pick C1 NPC1
AIPGENE721	3.0	6E-11	Sialate O-acetylesterase
AIPGENE19216	2.5	2E-23	Acid ceramidase
AIPGENE1519	2.5	5E-19	Tyrosine-protein kinase SRC42A
AIPGENE9971	2.5	2E-19	Lysosomal aspartic protease
AIPGENE26157	2.2	7E-13	Cathepsin L
AIPGENE22539	2.2	1E-05	Epididymal secretory protein NPC2
AIPGENE3899	2.0	2E-11	CD63 antigen
AIPGENE756	2.0	4E-30	Putative phospholipase B-like 2
AIPGENE5532	2.0	3E-07	Niemann-Pick C1 NPC1
AIPGENE18432	2.0	4E-21	Low-density lipoprotein receptor-related protein 6

[a] Ordered by Fold change.

[b] Fold-changes calculated by comparing the cells in Cluster 10 with all the other cells.

Table S14 Cluster 10 marker genes associated with glucose transport and its positive regulation

Gene ID[a,b]	Fold change[c]	Adjusted *p* value	Annotation
AIPGENE12082	1.8	4E-24	Solute carrier family 2, facilitated glucose transporter member 1
AIPGENE10547	1.5	2E-25	C2 domain-containing protein 5
AIPGENE10700	1.3	4E-04	Bifunctional protein NCOAT

[a] Ordered by Fold change.

[b] Gray highlight, the gene encoding glucose transporter AipGLUT1α, as investigated further in this study.

[c] Fold-changes calculated by comparing the cells in Cluster 10 with all the other cells.

Table S15 Cluster 10 marker genes associated with cholesterol transport and homeostasis

Gene ID[a]	Fold change[b]	Adjusted *p* value	Annotation
AIPGENE5531	3.3	3E-21	Niemann-Pick C1 NPC1
AIPGENE22539	2.2	9E-06	Epididymal secretory protein NPC2
AIPGENE5532	2.0	3E-07	Niemann-Pick C1 NPC1
AIPGENE18432	2.0	4E-21	Low-density lipoprotein receptor-related protein 6

[a] Ordered by Fold change.

[b] Fold-changes calculated by comparing the cells in Cluster 10 with all the other cells.

Table S16 Cluster 7 marker genes associated with extracellular matrix, cell adhesion, and cell-cell signaling

Gene ID[a]	Fold change[b]	Adjusted *p* value	Annotation
AIPGENE10422	18	1E-85	Collagen alpha-1(IV) chain
AIPGENE10546	11	4E-65	Collagen alpha-2(IV) chain
AIPGENE8588	9.0	2E-60	Testican-2
AIPGENE1266	8.2	6E-28	Somatomedin-B and thrombospondin type-1 domain-containing protein
AIPGENE6165	8.2	5E-22	Integrin alpha-8
AIPGENE27343	7.4	2E-46	Collagen alpha-4(VI) chain
AIPGENE23790	6.0	3E-60	Integrin beta-6
AIPGENE2489	6.0	6E-34	Matrilin-2
AIPGENE3595	5.5	1E-32	Serotransferrin
AIPGENE9466	5.5	8E-16	Fibroblast growth factor 18
AIPGENE7597	5.0	7E-46	Zonadhesin
AIPGENE1737	5.0	6E-44	Protein Wnt
AIPGENE23312	5.0	1E-39	Hemicentin-1
AIPGENE12342	5.0	4E-28	Collagen alpha-3(VI) chain
AIPGENE22492	5.0	7E-37	Glypican-6
AIPGENE7897	5.0	2E-15	Hemicentin-1
AIPGENE23262	4.5	6E-56	Fibroblast growth factor receptor 1
AIPGENE17918	4.5	2E-16	Hemicentin-2
AIPGENE10250	4.5	6E-21	Collagen alpha-6(VI) chain
AIPGENE20628	4.5	6E-47	Collagen alpha-4(VI) chain
AIPGENE23383	4.1	5E-38	Hemicentin-2
AIPGENE29129	4.1	6E-40	Periostin
AIPGENE6975	4.1	8E-43	Neurofascin
AIPGENE16436	4.1	5E-53	Protocadherin Fat 1
AIPGENE27342	4.1	2E-07	Agrin
AIPGENE11184	4.1	3E-16	Collagen alpha-1(VII) chain
AIPGENE17222	3.7	3E-29	Agrin
AIPGENE25153	3.7	6E-24	Collagen alpha-5(VI) chain
AIPGENE10561	3.7	4E-17	Collagen alpha-1(XXVI) chain
AIPGENE21255	3.7	2E-12	Integrin alpha-4
AIPGENE12362	3.3	8E-08	Collagen alpha-6(VI) chain
AIPGENE20627	3.3	3E-09	Collagen alpha-3(VI) chain
AIPGENE7612	3.3	2E-26	Reticulocyte-binding protein 2 homolog a
AIPGENE8011	3.0	5E-15	SCO-spondin
AIPGENE16749	3.0	3E-11	Talin-2
AIPGENE18950	3.0	1E-06	Metastasis suppressor protein 1
AIPGENE3608	3.0	3E-07	Receptor-type tyrosine-protein phosphatase delta
AIPGENE12347	3.0	7E-15	Collagen alpha-6(VI) chain
AIPGENE266	3.0	1E-10	Dystroglycan

AIPGENE23512	3.0	1E-06	Protein Wnt-4
AIPGENE3034	2.7	2E-24	Mammalian ependymin-related protein 1
AIPGENE8440	2.7	1E-11	Integrin alpha-6
AIPGENE29194	2.7	3E-14	Catenin delta-2
AIPGENE4075	2.5	4E-14	Proprotein convertase subtilisin/kexin type 5
AIPGENE15963	2.5	1E-11	Laminin subunit beta-1
AIPGENE14892	2.5	9E-16	Calsyntenin-2
AIPGENE26184	2.5	7E-11	Fibrillin-1
AIPGENE9673	2.5	6E-10	Fibulin-1
AIPGENE22521	2.2	9E-10	Glypican-1
AIPGENE25192	2.2	8E-22	Collagen alpha-1(XXIV) chain
AIPGENE15522	2.2	2E-06	Protocadherin Fat 1
AIPGENE17012	2.2	3E-12	Laminin subunit alpha-4
AIPGENE19606	2.2	2E-05	Thrombospondin-1
AIPGENE18534	2.2	1E-20	Collagen alpha-2(I) chain
AIPGENE5847	2.2	8E-07	Semaphorin-5A
AIPGENE25893	2.2	3E-11	Collagen alpha-1(XXVII) chain B
AIPGENE8276	2.0	2E-08	Myosin-10
AIPGENE18348	2.0	4E-05	Fibrillin-1
AIPGENE8376	2.0	6E-12	Collagen alpha-2(I) chain
AIPGENE2639	2.0	5E-06	Afadin
AIPGENE6961	2.0	2E-10	Neurofascin
AIPGENE2368	2.0	5E-06	Laminin subunit gamma-1

[a] Ordered by Fold change.

[b] Fold-changes calculated by comparing the cells in Cluster 7 with all the other cells.

Table S17 Expression patterns of the five major glucose and ammonium transporters

Gene[a]	Average expression level (TPM)[b]		Fold change[b]	
	SymEpi	SymGas	SymEpi/ApoEpi	SymGas/ApoGas
AipSLC2A8α (AIPGENE2706)	2.2	21	0.84	4.7
AipGLUT1α (AIPGENE12082)	33	174	0.67	1.0
AipGLUT8α (AIPGENE18406)	8.9	70	2.1	6.8
AipRhBG1(AIPGENE18105)	20	410	6.8	170
AipAMT1 (AIPGENE17420)	98	25	1.1	1.4

[a] Genes are listed in the order in which they are shown in Figure 2 A, B, and E – S.

[b] Numbers are rounded to two significant digits.

Table S18 Peptides used as antigens to generate custom antibodies

Target ID	Peptide sequence[a,b]
AipAMT1 (AIPGENE17420)	NGGSQGSIASKGDA
AipRhBG1 (AIPGENE18105)	IDQESGHSINSIKS
AipSLC2A8α (AIPGENE2706)	ALLGGPLGGWLIEAFGRKGG
AipGLUT1α (AIPGENE12082)	NSPEKVIKDYYKKYGHEFTD
AipGLUT8α (AIPGENE18406)	PDMDIDFEIA

[a] Peptides were selected from non-transmembrane regions with high predicted antigenicity and hydrophilicity.

[b] An additional cysteine was added to either the N- or C-terminus of each peptide to facilitate its conjugation to keyhole-limpet hemocyanin to create the antigens used for immunization.

Table S19 Expression levels of genes associated with "nitrogen-metabolism pathway" (KEGG Ko00910) in Aiptasia

Gene ID[a,b]	TPM			Annotation
	Apo	Apo + Gluc	Sym	
AIPGENE510	360	240	300	Cystathionine gamma-lyase
AIPGENE17420	26	360	270	Putative ammonium transporter 1
AIPGENE26763	60	310	244	Glutamine synthetase
AIPGENE24026	10	140	170	Glutamate dehydrogenase
AIPGENE18105	3.1	4.2	94	Ammonium transporter Rh type B
AIPGENE26078	7.7	47	68	Putative glutamate synthase [NADPH]
AIPGENE5302	86	51	56	Carbonic anhydrase 2
AIPGENE17478	25	50	48	Asparagine synthetase
AIPGENE28157	25	37	37	Carbamoyl-phosphate synthase
AIPGENE2960	190	140	35	Carbonic anhydrase 2
AIPGENE21724	35	48	33	Omega-amidase NIT2
AIPGENE10420	24	31	31	Carbonic anhydrase 7
AIPGENE659	19	22	25	vWA domain-containing protein 8
AIPGENE19720	40	18	19	Carbonic anhydrase 2
AIPGENE3776	28	19	17	Glutamate dehydrogenase, mitochondrial
AIPGENE19710	17	33	15	Carbonic anhydrase-related protein 10
AIPGENE7282	15	18	13	Ammonium transporter Rh type B
AIPGENE27618	28	10	9.8	Glutamate dehydrogenase
AIPGENE529	11	7.3	8.9	Cystathionine gamma-lyase
AIPGENE17792	15	6.6	8.7	Histidine ammonia-lyase
AIPGENE19737	1.6	2.6	5.4	Carbonic anhydrase 2
AIPGENE15516	2.7	3.6	4.8	Putative ammonium transporter 3
AIPGENE16683	5.3	3.2	3.5	Putative ammonium transporter 3
AIPGENE27316	3	4.4	2.8	Asparagine synthetase
AIPGENE15200	2.2	1.5	2.6	Putative ammonium transporter 3
AIPGENE878	2.8	7.4	1.9	Tryptophanase
AIPGENE19694	3.4	8	1.8	Carbonic anhydrase 2
AIPGENE16631	1.5	0.36	0.76	Putative ammonium transporter 3

[a] Ordered by expression level (TPM) in Sym
[b] Genes marked in Fig. 3B are highlighted in yellow

Table S20 Primers used to amplify the transporter genes

Target ID	Gene size	Forward primer	Reverse primer
AipAMT1 (AIPGENE17420)	1443 bp	atgatgaacgtaactacagaacagac	ttacaatttttcatcaagagctccgtg
AipRhBG1 (AIPGENE18105)	1501 bp	atgtgctcggcaatcataacaacacgc	ttaatcactcttgatggaattgatggaatgtcc
AipSLC2A8α (AIPGENE2706)	1431 bp	GCTAGCatggccaatcaaaatattcaaagtagc	GGATCCttatatgcgttcatattcag
AipGLUT1α (AIPGENE12082)	1470 bp	GCTAGCatggaggaggaaaagggggagac	GGATCCttaatcatcgtccccaacagaac
AipGLUT8α (AIPGENE18406)	1476 bp	GCTAGCatgaatcgttcattactcaagcg	GGATCCttaaagtctgctttctacctctgc

Sequences in uppercase indicate the enzyme-cutting sites for *Nhe*I (Forward primer) and *Bam*HI (Reverse primer), used in cloning the amplified products into plasmid pSH100 (see text). Gene-specific-primer sequences are in lowercase. No enzyme-cutting sites were added for the ammonium-transporter genes, as they were cloned using a TA-cloning protocol.

Data S1 Tissue-specific transcriptomic profiles of Aiptasia in different symbiotic states. Gene-expression levels are reported as transcripts per million (TPM). Apo, aposymbiotic; Sym, symbiotic; Epi, epidermis; Gas, gastrodermis.

Data S2 Differentially expressed genes identified from multi-factor analysis. Numbers represent z-scores calculated based on gene-expression levels (TPM). Modules were assigned using hierarchical clustering. Apo, aposymbiotic; Sym, symbiotic; Epi, epidermis; Gas, gastrodermis.

Data S3 Potential cell-type markers identified in single-cell RNA-seq.

Data S4 Gene-set-enrichment analysis of identified Cluster 10 marker genes.

Data S5 Gene-set-enrichment analysis of identified Cluster 7 marker genes.
